# 7-Alkoxy-appended coumarin derivatives: synthesis, photo-physical properties, aggregation behaviours and current–voltage (*I*–*V*) characteristic studies on thin films†

**DOI:** 10.1039/d1ra00762a

**Published:** 2021-03-10

**Authors:** Abhijit Rudra Paul, Bapi Dey, Sudip Suklabaidya, Syed Arshad Hussain, Swapan Majumdar

**Affiliations:** Department of Chemistry, Tripura University Suryamaninagar 799 022 India smajumdar@tripurauniv.in +91-381-2374802 +91-381-237-9070; Department of Physics, Tripura University Suryamaninagar 799 022 India

## Abstract

In this study, we designed and synthesised a series of coumarin derivatives appended with a long alkoxy chain on the seventh position of the coumarin-3-carboxylate/carboxylic acid core to make thin film materials. Synthesised compounds were characterized by their UV and fluorescence spectra in solutions as well as their films prepared by both LB and spin-coated methods. The surface morphology and electrical behaviour of thin films were judged by AFM, SEM and *I*–*V* characteristic mapping respectively. Isotherm, UV-Vis absorption and fluorescence spectroscopic investigations revealed the formation of aggregates on thin films. The result of SEM and AFM images provides the information about the length and height of aggregates on the thin films of coumarin derivatives. From *I*–*V* characteristics, it was found that at room temperature, the spin-coated films of coumarin derivatives containing an ester functional group exhibited a threshold switching behaviour, whereas derivatives containing the carboxylic acid functional group showed both threshold and bipolar switching behaviours. We also noticed that the *I*–*V* characteristic features of synthesized materials depended on the length of the alkyl chain of individual series.

## Abstract 2

In this article, we demonstrate the design, synthesis and physico-chemical characteristics, including electrical switching behaviours of long alkoxy-appended coumarin carboxylate/carboxylic acid in thin films.

## Graphical abstract

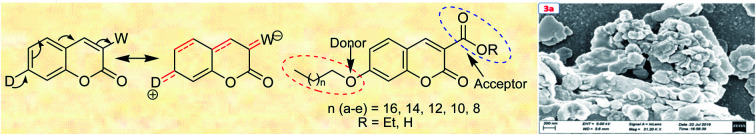

## Introduction

Electronic and optoelectronic devices are used in various applications ranging from simple household appliances to communications, computing, and medical instruments, and therefore, they are considered as basic building blocks for modern civilization. Given the demand for ever more compact and powerful systems, there is growing interest in the development of nanoscale devices that could enable new functions and/or greatly enhance the performance. During the last decade, organic electronics have been widely adopted in various electrical, electronic and optoelectronic applications.^1^ They provide alternative options in terms of the ease of processing, low cost, better flexibility, and superior electronic/optoelectronic properties.^2^ Taking the advantage of solution-processing, self-assembly and surface-engineering, organic materials could serve as new building blocks for the low-cost manufacturing of flexible and large-area devices.^3^ Organic electronics deals with various interesting organic materials and these innovative organic nanomaterials exhibit a variety of important properties such as optical, electrical, photoelectrical conducting, semiconducting, memory, storage and magnetic properties.^4^ Recent extensive studies have shown that organic materials have distinctive advantages such as the low cost of processing, lightweight, mechanical flexibility, ambient processing, printability, and a variety of materials, which have attracted specific attention towards organic electronics.^5^ In addition, several organic materials have been used as principal components to design various electronic and optoelectronic devices such as diodes, sensors, organic light-emitting diodes (OLED), organic field effect transistors (OFET), solar cells, lasers, detectors, memory-switching devices, and logic gates.^6^ Nowadays, molecular electronics has emerged as an important technology. It deals with the design, processing and device application of organic molecules at the molecular level/nanoscale level.^7^ Thin-film-fabricated switching devices of organic molecules are promising candidates for the next generation of non-volatile memories due to their simple structure, low cost, excellent performance and great scale-down potential.^8^

**Fig. 1:**
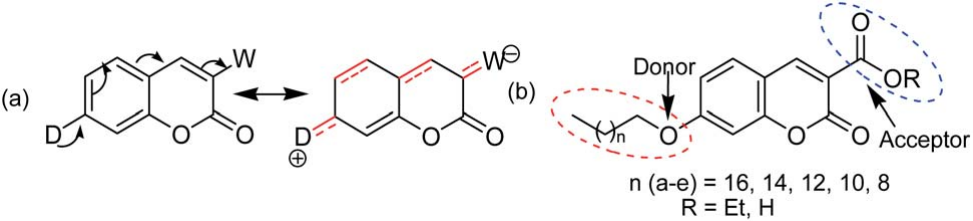
(a) Intramolecular charge transfer and (b) general structures of the synthesized coumarin derivatives 2a–e (R = Et) and 3a–e (R = H) used in the present study.

Coumarins structurally belong to a lactone family constructed with a benzene ring fused to an α-pyrone ring and essentially possess a conjugated system with electron rich and good charge transport properties.^9^ The structural simplicity and versatility of the coumarin scaffold make them interesting starting point for a wide range of applications. They have been found to show versatile pharmacological and biological activities,^10–15^ which can display anti-cancer, anti-HIV, anti-tumor, anti-microbial, antibacterial, anti-inflammatory, anti-oxidant, anti-diabetic, anti-coagulant functions, *etc.* Despite tremendous biological activities of coumarin derivatives, only very limited studies have been undertaken on their photo-physical and spectroscopic studies.^16–18^ The fluorescence of coumarin can be tuned by introducing different substituents with varied electron-donating/withdrawing abilities, which is widely used in the design of coumarin derivatives. The substituent-dependent fluorescence properties of the coumarin moiety may result from intramolecular charge transfer.^19,20^ It is well known^21^ that π–π* transitions can form the excited states *via* excitation and usually transitions of coumarin are related to the intra-molecular charge transfer from the benzene ring to the pyranone moiety. Thus, an electron-donating group at position 7 and/or an electron-withdrawing group at position 3 can promote the process, thereby enhancing the fluorescence intensity of coumarin [shown in Fig. 1(a)]. Coumarins substituted at position 7 with an electron-donating group are known to exhibit strong fluorescence as optical brighteners, solar energy collectors, laser dyes and fluorescent probes.^22–28^ Accordingly this type of photo and electro-active organic materials have been the subject of current research including organic semiconductors, organic metals including superconductors, organic photoconductors, organic photoactive materials for solar cells, organic non-linear optical materials, and liquid crystals.^29–32^ In this respect, the *I*–*V* characteristics for resistive bipolar and threshold switching in thin films are very important due to their promising application as active components for the memory device, logic circuits, *etc.*, to realize the molecular electronic structure.^33,34^ To this end, five and six-membered heterocycles, in particular coumarins, seem to be suitable parent π-conjugated backbones as they represent a robust and stable heterocycle, and can easily be synthesized in high purities and further functionalized at suitable positions by a simple chemistry to make them useful for devices. Given the advantages of small molecules of well-defined molecular structures, it is worthwhile to explore the physicochemical behaviour of novel coumarin derivatives. In this work, the synthesis, and spectroscopic, morphological and electrical behaviours *via I*–*V* characteristic mapping of a series of coumarin derivatives appended with a long alkoxy chain at the C-7 position of coumarin-3-carboxylates/carboxylic acids [Fig. 1(b)] are reported. The alkoxy group is chosen because it can act as a donor as well as help in aggregation for making thin films *via* hydrophobic–hydrophobic interactions, and electron-withdrawing groups at C-3 can modulate the electronic behaviour of coumarin derivatives. Our study reveals that the aggregation behaviour depends on the length of the alkyl chain as well as the nature of the group at C-3. Electrical switching behaviours *via I*–*V* characteristic mapping of spin-coated films of coumarin derivatives containing an ester group at C-3 show threshold switching, whereas a carboxylic acid group shows both threshold and bipolar memory switching. *I*–*V* characteristic features also depend on the length of the alkyl chain of individual series.

## Experimental section

### General

NMR spectra were recorded using a Bruker Ascend 400 spectrophotometer. ^1^H NMR and ^13^C NMR spectra were recorded at ambient temperature using 400 MHz spectrometers (400 MHz for ^1^H and 100 MHz for ^13^C). Chemical shifts are expressed in parts per million from the tetramethylsilane internal reference, and coupling constants are expressed in Hertz. Proton multiplicities are represented as s (singlet), d (doublet), dd (double doublet), t (triplet), q (quartet), and m (multiplet). Infrared spectra were recorded using a Fourier transform infrared (FT-IR, Model: Spectrum 100) spectrometer with KBr pellets for thin films. UV-vis absorption and fluorescence spectra of pure solutions as well as spin-coated films in quartz slide were recorded using a Perkin Elmer Lambda-25 Spectrophotometer and Perkin Elmer LS-55 fluorescence spectrophotometer respectively. A commercially available Langmuir–Blodgett (LB) film deposition instrument (Apex 2006C, Apex Instruments Co., India) was used for isotherm measurement. The spin-coated films were prepared onto ITO-coated glass slides using a spin coating film deposition instrument SUC2005A (Apex instrument company). Spin-coated films of study materials were prepared using a spin coater unit, Model: EZ spin-SD, Apex Instruments Co., India. An atomic force microscopic (AFM) image in the intermittent contact (tapping) mode of spin-coated films was taken in air (AFM system: Bruker Innova). The typical scan area was different for different compounds. A silicon-wafer substrate was used for the AFM measurement. The surface morphology of the aggregate was estimated using a Field Emission Scanning Electron Microscope Model – Sigma 300, Carl Zeiss instrument. The current–voltage (*I*–*V*) characteristics were measured using a Keithley 2401 source meter. The reported melting points were uncorrected. High-resolution mass spectrometry (HR-MS) data were acquired by electron spray ionization using a Q-ToF-micro quadrupole mass spectrometer.

### Materials

All reagents were purchased from commercial suppliers and used without further purification, unless otherwise specified. Commercially supplied ethyl acetate and petroleum ether (60–80 °C) were distilled before use. Column chromatography was performed on silica gel (60–120 mesh, 0.12–0.25 mm). Analytical thin-layer chromatography (TLC) was performed on 0.25 mm extra-hard silica gel plates with a UV254 fluorescent indicator. 2,4-Dihydroxy benzaldehyde was purchased from Sigma Aldrich chemical Co., USA, and used as received. Diethyl malonate, anhydrous K_2_CO_3_, and DMF were purchased from SRL India. Dry CH_2_Cl_2_ and dry DMF were prepared according to the standard procedures. Spectroscopy-grade chloroform and ultra-pure Milli-Q water (triple distilled deionized resistivity = 18.2 MΩ cm) were used throughout the study. The synthesized product was characterized by melting point, IR data, ^1^H NMR, ^13^C NMR and mass spectral analysis.

### Synthesis

#### Synthesis of ethyl 7-hydroxy-2-oxo-chromene-3-carboxylate (1)

A mixture of 2,4-dihydroxybenzaldehyde (1 g, 7.194 mmol), diethyl malonate (1.31 mL or 1.38 g, 8.615 mmol), absolute ethanol (5 mL), piperidine (15 drops) followed by glacial acetic acid (5 drops) was heated at reflux at 80 °C for 1.5 h. After the completion of reaction (TLC), the reaction mixture was allowed to cool below 60 °C and then water (15 mL) was added to the flask. The reaction mixture was then chilled in an ice bath. The solid product thus obtained was collected by filtration, washed with a small amount of chilled 50% aqueous ethanol and dried. The analytical sample was prepared by crystallization from water–ethanol. Yield: 85%, white solid, mp 168–170 °C; lit.^35^ mp 166–168 °C; IR (KBr) *ν*_max_ 3542, 2817, 1736, 1612, 1531, 1450, 1226, 1032 cm^−1^; ^1^H NMR (400 MHz, CDCl_3_) *δ* 11.14 (s, 1H), 8.66 (s, 1H), 7.74 (d, *J* = 8.8 Hz, 1H), 6.80 (t, *J* = 8.4 Hz, 2H), 4.25 (q, *J* = 7.2 Hz, 2H), 1.29 (t, *J* = 7.2 Hz, 3H); ^13^C NMR (100 MHz, CDCl_3_) *δ* 164.5, 163.4, 157.5, 156.8, 149.9, 132.5, 114.4, 112.5, 110.8, 102.2, 61.3, 14.5.

#### General procedure for the synthesis of 2a–e by alkylation of ethyl 7-hydroxy-2-oxo-chromene-3-carboxylate

A mixture of ethyl 7-hydroxy-2-oxo-chromene-3-carboxylate (1, 1 g, 4.27 mmol), long-chain alkyl bromide (C_*n*_H_2*n*+2_Br, *n* = 18, 16, 14, 12 and 10; 5.13 mmol), anhydrous potassium carbonate (8.54 mmol) and DMF (5 mL) was stirred at 60 °C for 1.5 h. After completion of the reaction (TLC), the reaction mixture was diluted with water (30 mL) and extracted with ethyl acetate (3 × 10 mL). The combined organic layer was washed with water (3 × 10 mL), dried over anhydrous sodium sulphate and concentrated under reduced pressure. The crude product was purified over silica-gel (60–120 mesh) using ethyl acetate–hexane (3 : 7) as the eluent.

#### Ethyl 7-octadecyloxy-2-oxo-2*H*-chromene-3-carboxylate (2a)

Yield: 84%; white solid, mp 76–78 °C; UV (CHCl_3_) *λ*_max_ 353 nm; IR (KBr) *ν*_max_ 2918, 2850, 1758, 1624, 1572, 1471, 1417, 1292, 1226 cm^−1^; ^1^H NMR (400 MHz, CDCl_3_) *δ* 8.50 (s, 1H), 7.47 (d, *J* = 8.4 Hz, 1H), 6.87 (dd, *J* = 2.0, 8.4 Hz, 1H), 6.78 (d, *J* = 2.0 Hz, 1H), 4.38 (q, *J* = 7.2, 14.4 Hz, 2H), 4.03 (t, *J* = 6.4 Hz, 2H), 1.84–1.78 (m, 2H), 1.47–1.41 (m, 2H), 1.39 (t, *J* = 7.2 Hz, 3H), 1.25 (bs, 28H), 0.87 (t, *J* = 6.4 Hz, 3H); ^13^C NMR (100 MHz, CDCl_3_) *δ* 164.7, 163.5, 157.6, 157.2, 149.0, 130.6, 114.0, 113.8, 111.4, 100.7, 68.6, 61.7, 31.9, 29.7, 29.5, 29.4, 29.3, 29.2, 28.8, 25.9, 22.7, 14.2, 14.1; HRMS calcd for C_30_H_46_O_5_ + H^+^ (M + H^+^) 487.3425; found 487.3466.

#### Ethyl 7-hexadecyloxy-2-oxo-2*H*-chromene-3-carboxylate (2b)

Yield: 88%; white solid, mp 80–82 °C; UV (CHCl_3_) *λ*_max_ 353 nm; IR (KBr) *ν*_max_ 2917, 2851, 1752, 1627, 1578, 1416, 1262, 1242, 1022 cm^−1^; ^1^H NMR (400 MHz, CDCl_3_) *δ* 8.52 (s, 1H), 7.50 (d, *J* = 8.8 Hz, 1H), 6.88 (dd, *J* = 2.0, 8.8 Hz, 1H), 6.80 (d, *J* = 2.4 Hz, 1H), 4.40 (q, *J* = 7.2, 14.4 Hz, 2H), 4.04 (t, *J* = 6.4 Hz, 2H), 1.86–1.79 (m, 2H), 1.47–1.45 (m, 2H), 1.40 (t, *J* = 7.2 Hz, 3H), 1.26 (bs, 24H), 0.88 (t, *J* = 6.8 Hz, 3H); ^13^C NMR (100 MHz, CDCl_3_) *δ* 164.8, 163.5, 157.6, 157.3, 149.1, 130.7, 114.1, 113.8, 111.4, 100.8, 69.0, 61.7, 31.9, 29.7, 29.6, 29.5, 29.4, 29.3, 28.9, 25.9, 22.7, 14.3, 14.1; HRMS calcd for C_28_H_42_O_5_ + H^+^ (M + H^+^) 459.3112; found 459.3103.

#### Ethyl 7-tetradecyloxy-2-oxo-2*H*-chromene-3-carboxylate (2c)

Yield: 89%; white solid, mp 74–76 °C; UV (CHCl_3_) *λ*_max_ 353 nm; IR (KBr) *ν*_max_ 2917, 2844, 1757, 1612, 1578, 1412, 1294, 1214, 1141 cm^−1^; ^1^H NMR (400 MHz, CDCl_3_) *δ* 8.52 (s, 1H), 7.51 (d, *J* = 8.4 Hz, 1H), 6.88 (dd, *J* = 2.4, 8.8 Hz, 1H), 6.80 (d, *J* = 2.4 Hz, 1H), 4.40 (q, *J* = 7.2, 14.4 Hz, 2H), 4.04 (t, *J* = 6.8 Hz, 2H), 1.86–1.79 (m, 2H), 1.47–1.45 (m, 2H), 1.41 (t, *J* = 7.2 Hz, 3H), 1.27 (bs, 20H), 0.89 (t, *J* = 6.8 Hz, 3H); ^13^C NMR (100 MHz, CDCl_3_) *δ* 164.8, 163.5, 157.6, 157.3, 149.1, 130.7, 114.1, 113.8, 111.4, 100.8, 69.0, 61.7, 31.9, 29.7, 29.6, 29.5, 29.4, 29.3, 28.88, 25.9, 22.7, 14.3, 14.1; HRMS calcd for C_26_H_38_O_5_ + H^+^ (M + H^+^) 431.2799; found 431.2701.

#### Ethyl 7-dodecyloxy-2-oxo-2*H*-chromene-3-carboxylate (2d)

Yield: 88%; white solid, mp 64–66 °C; UV (CHCl_3_) *λ*_max_ 353 nm; IR (KBr) *ν*_max_ 1756, 1641, 1566, 1418, 1348, 1220, 1018 cm^−1^; ^1^H NMR (400 MHz, CDCl_3_) *δ* 8.53 (s, 1H), 7.51 (d, *J* = 8.4 Hz, 1H), 6.89 (dd, *J* = 2.4, 8.8 Hz, 1H), 6.81 (d, *J* = 2.4 Hz, 1H), 4.41 (q, *J* = 7.2, 14.4 Hz, 2H), 4.05 (t, *J* = 6.4 Hz, 2H), 1.85–1.82 (m, 2H), 1.47–1.45 (m, 2H), 1.42 (t, *J* = 7.2 Hz, 3H), 1.28 (bs, 16H), 0.89 (t, *J* = 6.4 Hz, 3H); ^13^C NMR (100 MHz, CDCl_3_) *δ* 164.7, 163.5, 157.6, 157.3, 149.1, 130.6, 114.0, 113.7, 111.4, 100.7, 68.9, 61.7, 31.8, 29.6, 29.5, 29.4, 29.3, 28.8, 25.9, 22.7, 14.3, 14.1; HRMS calcd for C_24_H_34_O_5_ + H^+^ (M + H^+^) 403.2484; found 403.2370.

#### Ethyl 7-decyloxy-2-oxo-2*H*-chromene-3-carboxylate (2e)

Yield: 97%; white solid, mp 58 °C; UV (CHCl_3_) *λ*_max_ 353 nm; IR (KBr) *ν*_max_ 1750, 1642, 1560, 1419, 1345, 1300, 1213 cm^−1^; ^1^H NMR (400 MHz, CDCl_3_) *δ* 8.52 (s, 1H), 7.50 (d, *J* = 8.8 Hz, 1H), 6.88 (dd, *J* = 2.4, 8.8 Hz, 1H), 6.81 (d, *J* = 2.0 Hz, 1H), 4.41 (q, *J* = 7.2, 14.4 Hz, 2H), 4.05 (t, *J* = 6.8 Hz, 2H), 1.85–1.80 (m, 2H), 1.48–1.45 (m, 2H), 1.41 (t, *J* = 7.2 Hz, 3H), 1.28 (bs, 12H), 0.89 (t, *J* = 6.4 Hz, 3H); ^13^C NMR (100 MHz, CDCl_3_) *δ* 164.8, 163.5, 157.6, 157.3, 149.1, 130.7, 114.1, 113.8, 111.4, 100.8, 69.0, 61.7, 31.9, 29.5, 29.3, 28.9, 25.9, 22.7, 14.3, 14.1; HRMS calcd for C_22_H_30_O_5_ + H^+^ (M + H^+^) 375.2173; found 375.2046.

#### General procedure for the synthesis of coumarin 3-carboxylic acid derivatives 3a–e

To a solution of alkylated coumarin 3-carboxylate (2a–e, 2.06 mmol) in ethanol (5.0 mL), 10% aq. KOH solution (10 mL) was added at once. The flask was then fitted with a condenser and the solution was heated in a water bath at 80 °C for 30 minutes. Cold HCl (6 M) was added to the reaction mixture under ice cold conditions for the precipitation of the product. The product was then collected by filtration, washed thoroughly with cold water, dried and finally crystallized from ethanol–water. Identities of the compounds were established by IR data, ^1^H NMR, ^13^C NMR and mass spectral analysis.

#### Ethyl 7-octadecyloxy-2-oxo-2*H*-chromene-3-carboxylic acid (3a)

Yield: 99%; white solid, mp 120–122 °C; UV (CHCl_3_) *λ*_max_ 357 and 374 nm; IR (KBr) *ν*_max_ 2917, 2849, 1735, 1692, 1622, 1508, 1428, 1384, 1258, 1144, 815 cm^−1^; ^1^H NMR (400 MHz, CDCl_3_) *δ* 12.21 (bs, 1H), 8.85 (s, 1H), 7.62 (d, *J* = 8.8 Hz, 1H), 7.00 (dd, *J* = 2.0, 8.8 Hz, 1H), 6.90 (d, *J* = 2.0 Hz, 1H), 4.08 (t, *J* = 6.4 Hz, 2H), 1.88–1.81 (m, 2H), 1.47–1.43 (m, 2H), 1.25 (bs, 28H), 1.03 (t, *J* = 6.4 Hz, 3H); ^13^C NMR (100 MHz, CDCl_3_) *δ* 165.9, 164.6, 163.2, 157.1, 151.2, 131.6, 115.5, 112.1, 110.6, 101.2, 69.4, 31.9, 29.7, 29.6, 29.5, 29.3, 29.2, 28.8, 25.9, 22.7, 14.1; HRMS calcd for C_28_H_42_O_5_ + H^+^ (M + H^+^) 459.3112; found 459.3110.

#### Ethyl 7-hexadecyloxy-2-oxo-2*H*-chromene-3-carboxylic acid (3b)

Yield: 90%; white solid, mp 124 °C; UV (CHCl_3_) *λ*_max_ 357 and 374 nm; IR (KBr) *ν*_max_ 2917, 2851, 1752, 1627, 1578, 1416, 1262, 1242 cm^−1^; ^1^H NMR (400 MHz, CDCl_3_) *δ* 12.24 (bs, 1H), 8.87 (s, 1H), 7.65 (d, *J* = 8.8 Hz, 1H), 7.02 (dd, *J* = 2.4, 8.8 Hz, 1H), 6.92 (d, *J* = 2.0 Hz, 1H), 4.10 (t, *J* = 6.4 Hz, 2H), 1.90–1.82 (m, 2H), 1.52–1.46 (m, 2H), 1.41 (t, *J* = 7.2 Hz, 3H), 1.26 (bs, 20H), 0.88 (t, *J* = 6.8 Hz, 3H); ^13^C NMR (100 MHz, CDCl_3_) *δ* 164.8, 163.5, 157.6, 157.3, 149.1, 130.7, 114.1, 113.8, 111.4, 100.8, 69.0, 61.7, 31.9, 29.7, 29.6, 29.5, 29.4, 29.3, 28.88, 25.9, 22.7, 14.3, 14.1; HRMS calcd for C_26_H_38_O_5_ + H^+^ (M + H^+^) 431.2799; found 431.2701.

#### Ethyl 7-tetradecyloxy-2-oxo-2*H*-chromene-3-carboxylic acid (3c)

Yield: 98%; white solid, mp 126 °C; UV (CHCl_3_) *λ*_max_ 357 and 374 nm; IR (KBr) *ν*_max_ 2910, 2844, 1751, 1625, 1572, 1413, 1254, 1201 cm^−1^; ^1^H NMR (400 MHz, CDCl_3_) *δ* 12.25 (bs, 1H), 8.87 (s, 1H), 7.64 (d, *J* = 8.8 Hz, 1H), 6.88 (dd, *J* = 2.4, 8.8 Hz, 1H), 6.80 (d, *J* = 2.4 Hz, 1H), 4.04 (t, *J* = 6.8 Hz, 2H), 1.90–1.82 (m, 2H), 1.52–1.45 (m, 2H), 1.41 (t, *J* = 7.2 Hz, 3H), 1.26 (bs, 20H), 0.88 (t, *J* = 6.8 Hz, 3H); ^13^C NMR (100 MHz, CDCl_3_) *δ* 164.8, 163.5, 157.6, 157.3, 149.1, 130.7, 114.1, 113.8, 111.4, 100.8, 69.0, 61.7, 31.9, 29.7, 29.6, 29.5, 29.4, 29.3, 28.88, 25.9, 22.7, 14.3, 14.1; HRMS calcd for C_24_H_34_O_5_ + H^+^ (M + H^+^) 403.2486; found 403.2488.

#### Ethyl 7-dodecyloxy-2-oxo-2*H*-chromene-3-carboxylic acid (3d)

Yield: 98%; white solid, mp 128–130 °C; UV (CHCl_3_) *λ*_max_ 357 and 374 nm; IR (KBr) *ν*_max_ 1641, 1562, 1414, 1348, 1018 cm^−1^; ^1^H NMR (400 MHz, CDCl_3_) *δ* 12.27 (bs, 1H), 8.88 (s, 1H), 7.63 (d, *J* = 8.8 Hz, 1H), 7.02 (d, *J* = 8.4 Hz, 1H), 6.93 (s, 1H), 4.11 (t, *J* = 6.4 Hz, 2H), 1.88–1.84 (m, 2H), 1.49 (bs, 2H), 1.28 (bs, 12H), 0.88 (t, *J* = 6.4 Hz, 3H); ^13^C NMR (100 MHz, CDCl_3_) *δ* 165.9, 164.7, 163.2, 157.1, 151.3, 131.7, 115.5, 112.2, 110.6, 101.2, 69.4, 31.9, 29.7, 29.6, 29.5, 29.4, 29.3, 28.8, 25.9, 22.7, 14.1; HRMS calcd for C_22_H_30_O_5_ + H^+^ (M + H^+^) 375.2173; found 375.1967.

#### Ethyl 7-decyloxy-2-oxo-2*H*-chromene-3-carboxylic acid (3e)

Yield: 91%; white solid, mp 132–134 °C; UV (CHCl_3_) *λ*_max_ 357 and 374 nm; IR (KBr) *ν*_max_ 1642, 1565, 1419, 1345, 1017, 930 cm^−1^; ^1^H NMR (400 MHz, CDCl_3_) *δ* 12.25 (bs, 1H), 8.87 (s, 1H), 7.65 (d, *J* = 8.8 Hz, 1H), 7.02 (dd, *J* = 2.4, 8.8 Hz, 1H), 6.92 (d, *J* = 2.4 Hz, 1H), 4.10 (t, *J* = 6.4 Hz, 2H), 1.90–1.83 (m, 2H), 1.53–1.45 (m, 2H), 1.36–1.29 (m, 12H), 0.90 (t, *J* = 6.4 Hz, 3H); ^13^C NMR (100 MHz, CDCl_3_) *δ* 165.9, 164.7, 163.2, 157.1, 151.3, 131.7, 115.5, 112.1, 110.6, 101.2, 69.4, 31.9, 29.5, 29.3, 28.8, 25.9, 22.7, 14.2; HRMS calcd for C_20_H_26_O_5_ + H^+^ (M + H^+^) 347.1860; found 347.1866.

## Results and discussions

### Synthesis

**Scheme 1:**
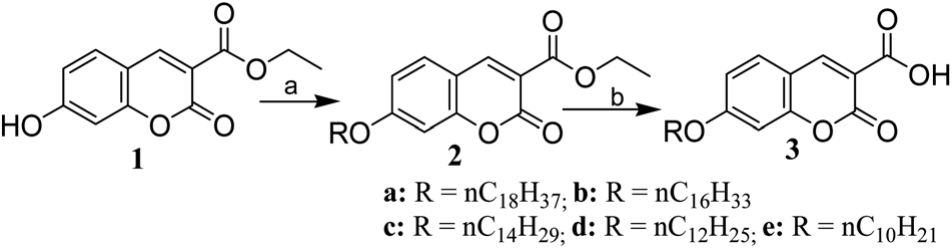
Synthesis of coumarin derivatives 2a–e and 3a–e. Reaction conditions: (a) anhydrous K_2_CO_3_, long-chain alkyl bromide (*n* = 18, 16, 14, 12 and 10), DMF, 60 °C, 1.5 h, 84–97%; (b) 10% aq. KOH, 80 °C, EtOH, 30 min, then 6 M HCl, 90–99%.

A series of coumarin derivatives (2a–e and 3a–e) containing a long alkoxy chain and an electron-withdrawing group at position 3 were prepared for our study by a straightforward two-step process from ethyl 7-hydroxy-coumarin-3-carboxylate (1), which was synthesized from 2,4-dihydroxybenzaldehyde and diethylmalonate by a known protocol.^35^ Treatment of 1 with alkyl bromide of variable chain length in the presence of anhydrous potassium carbonate in DMF afforded the corresponding *O*-alkylated products (2), which upon base-catalysed hydrolysis followed by acidification produced coumarin derivatives (3) in excellent yields (Scheme 1).

### UV-vis absorption spectroscopy measurement

**Fig. 2:**
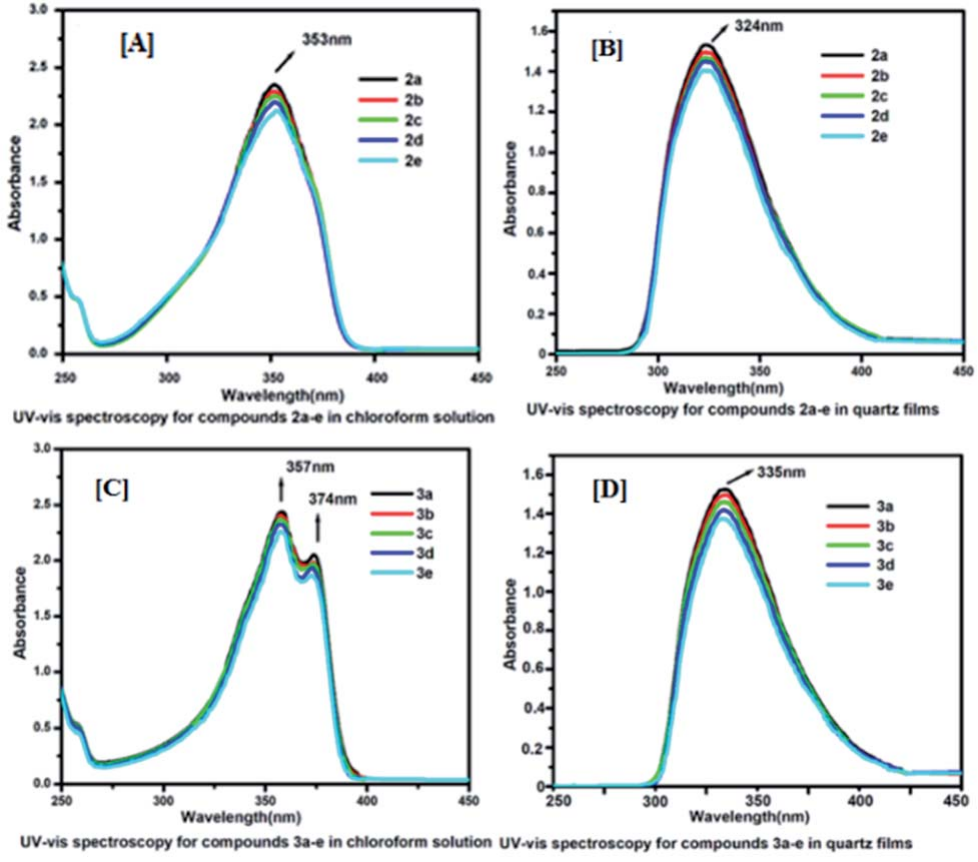
UV-Vis absorption spectra: Panels [A] and [B] for 2a–e in solutions and quartz films; Panels [C] and [D] for 3a–e in solutions and quartz films.

Initially, we began with the study of absorption spectroscopy of the synthesized molecules to understand their absorption behaviour in solutions as well as in thin films. Fig. 2[A] and [B] represents the absorption spectra of compounds 2a–e in CHCl_3_ solution (*c* 1.0 × 10^−5^ mol L^−1^) and spin-coated quartz films respectively. Compounds 2a–e exhibit a strong absorption band at 353 nm in the solution and at 324 nm in the spin-coated film. The absorption bands of spin-coated quartz films are blue-shifted with respect to the absorption bands of compounds (2a–e) in the chloroform solution. Similarly, compounds 3a–e in the solution (Fig. 2[C]) exhibit a strong absorption band at 357 nm with a weak shoulder at around 374 nm and in the spin-coated film show a absorption band at 335 nm (Fig. 2[D]). Compounds 3a–e also show a hypso-chromic shift (blue-shift) in spin-coated quartz films with respect to the solution absorption spectra. For both series, the absorption spectra of spin-coated films are shifted to a shorter wavelength or higher energy with respect to solution absorption bands. This kind of absorption shift indicated that in the film, the molecules were organized as H-aggregates *via* hydrophobic–hydrophobic interactions with long alkyl chains and π–π stacking between electron-rich coumarin cores. The intensities of absorption bands also depend on the length of the alkyl chain of the individual series.

### Fluorescence measurement

**Fig. 3:**
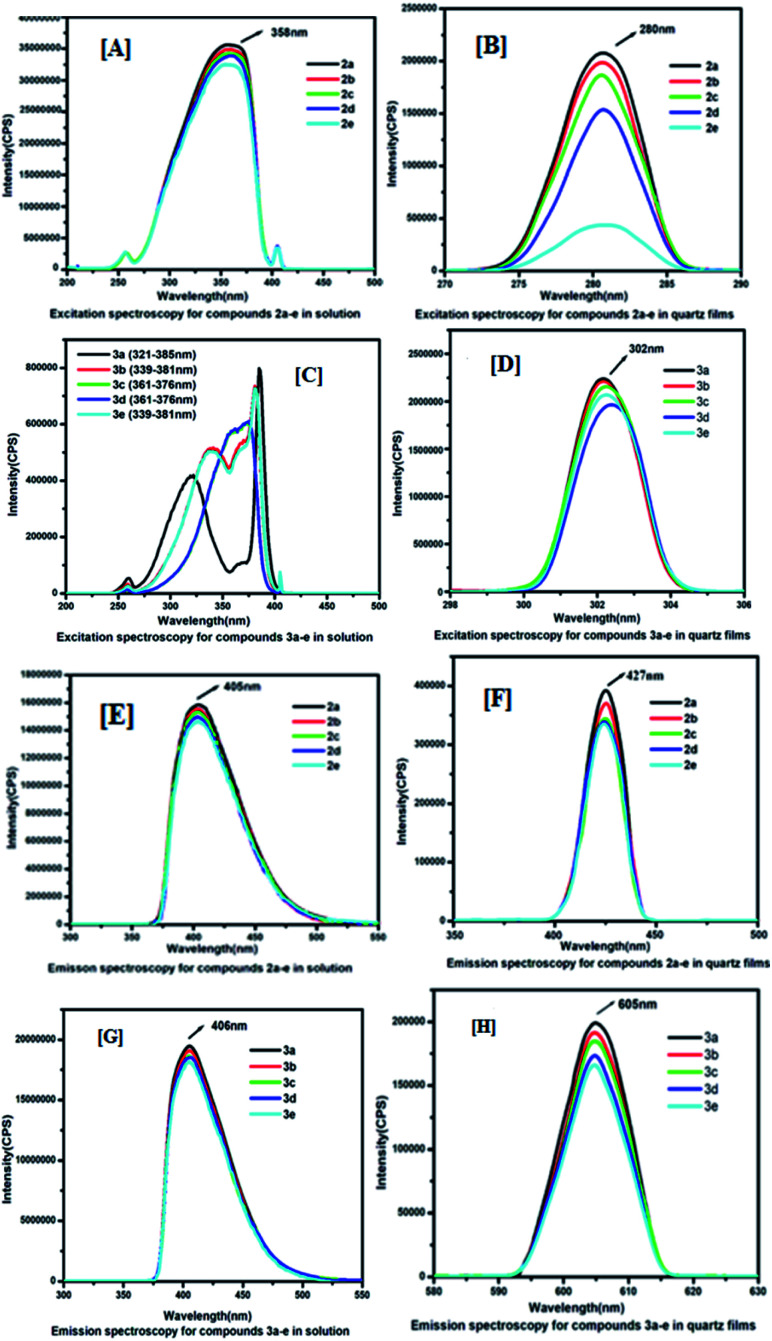
Fluorescence spectra for compounds (2a–e) and (3a–e): Panels [A], [B] and [C], [D] represents excitation spectra for compounds 2a–e and 3a–e in solutions and thin films respectively; Panels [E], [F] and [G], [H] represents emission spectra for compounds 2a–e and 3a–e in solutions and films respectively.

To understand the electronic excitation behaviour, we recorded the fluorescence spectra of compounds 2a–e and 3a–e in a chloroform solution at a concentration of 1.0 × 10^−5^ mol L^−1^ as well as in spin-coated thin films. The fluorescence spectra were recorded with excitation into the maximum of the longest wavelength absorption. Fig. 3[A]–[D] shows the excitation spectra of 2a–e and 3a–e in their solution and thin films. The excitation spectra were recorded for compounds 2a–e in solutions at 358 nm and the maximum absorption band at 353 nm, and for quartz films, it was excited at 280 nm and the absorption band appeared at 324 nm. Similarly, for compounds 3a–e, in the solution, the maximum excitation was recorded at 385 nm and maximum absorption band at 357 nm, and for quartz films, it was excited at 302 nm and the absorption band appeared at 335 nm. A longer excitation spectrum was recorded in solutions due to the presence of electron-withdrawing and -donating substituents that possess a strong push–pull system to exhibit high fluorescence intensities. The lower excitation of 3a–e in films may be attributed due to the compactness of the molecules *via* intermolecular hydrogen bonding between carboxylic acid functional groups.

The emission spectra showed significant dependence on the excitation wavelength. The emission spectra of 2a–e and 3a–e in the chloroform solution (1.0 × 10^−5^ mol L^−1^) and quartz film at different excitation wavelengths are displayed in Fig. 3[E]–[H]. The emission spectrum for compounds 2a–e obtained by excitation at 358 nm has an emission maximum of 405 nm in the chloroform solution, whereas in spin-coated films, the excitation wavelength is 280 nm and emission maximum is 427 nm. However, the emission spectrum for compounds 3a–e obtained by excitation at 385 nm has an emission maximum of 406 nm in the chloroform solution (1.0 × 10^−5^ mol L^−1^), whereas in quartz films, the excitation wavelength is 302 nm and the emission maximum is 605 nm. The fluorescence excitation and emission data of 2a–e and 3a–e are summarized in Table 1.

**Table 1:** UV-Vis absorption and fluorescence excitation/emission measurement in a chloroform solution (*c* 1.0 × 10^−5^ mol L^−1^) and a thin film

UV-Vis absorption/fluorescence excitation/emission	Compounds	
	2a–e	3a–e
UV-vis absorption maxima in solution	353 nm	357 nm
UV-vis absorption maxima of thin film	324 nm	335 nm
Fluorescence excitation maxima in solution	358 nm	385 nm
Fluorescence emission maxima in CHCl_3_ solution	405 nm	406 nm
Fluorescence excitation maxima of thin film (spin coated)	280 nm	302 nm
Fluorescence emission maxima of thin film (spin coated)	427 nm	605 nm

### Isotherm measurement/monolayer characteristics

**Fig. 4:**
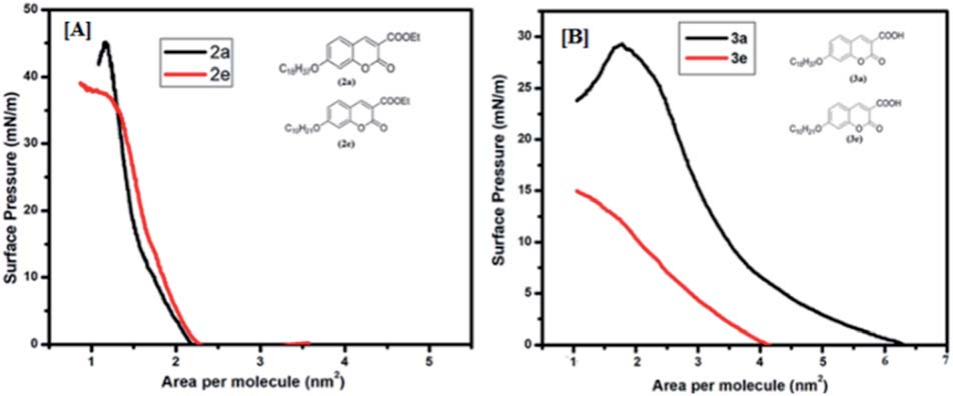
Surface pressure *vs.* area per molecule isotherms: [A] for (2a, 2e) and [B] for (3a, 3e). Inset shows the molecular structure of the compounds.

In order to have an idea about monolayer properties, two extreme members [2a (*n* = 16, R = Et), 2e (*n* = 8, R = Et), 3a (*n* = 16, R = COOH), and 3e (*n* = 8, R = COOH) in Fig. 1] of each series of the coumarin derivatives were selected and then surface pressure–area per molecule isotherm (*π*–*A*) at the air–water interface using the Langmuir–Blodgett (LB) techniques was recorded. A dilute solution in chloroform (1.0 × 10^−5^ mol L^−1^) of 100 μL of each material was spread by a micro syringe on the water sub-phase of pure Milli-Q water (18.2 MΩ cm) at room temperature and allowed to evaporate. After complete evaporation of the volatile solvent, the barrier was compressed at a rate of 5 mm min^−1^ to record the surface pressure–area per molecule isotherms. The surface pressure (*π*) *versus* average area (*A*) available for one molecule was measured by a Wilhelmy plate arrangement.^36^ Each isotherm was repeated a number of times and data for surface pressure-area per molecule isotherms were obtained by a computer interfaced with the LB instrument. Before each isotherm measurement, the trough and barrier were cleaned with ethanol and then rinsed with Milli-Q water. The compound (2a, 2e, 3a and 3e) monolayer formed at the air–water interface was found to be stable and easily transferable onto the solid substrate to monolayer LB films. Fig. 4[A] shows the corresponding (*π*–*A*) isotherm curves of compounds 2a and 2e. The isotherm of compound 2a starts rising with an initial lift off area of 2.2 nm^2^ and for compound 2e it was observed at 2.3 nm^2^. It was found that the isotherm of compound 2a gets collapsed at 45 mN m^−1^, whereas the collapse occurred at the lower surface pressure at 38 mN m^−1^ for compound 2e, *i.e.*2a forms a more stable film than 2e probably due to the presence of longer alkyl chains in 2a. However, isotherms of compounds 3a and 3e started rising with lift off areas 4.2 nm^2^ and 6.3 nm^2^ and gets collapsed after surface pressure 29 mN m^−1^ and 15 mN m^−1^ respectively (Fig. 4[B]). The isotherm profiles of compounds 3 (a, e) indicate a higher lift off area than the isotherm profiles of compounds 2 (a, e). The differences in lift off areas as well as collapse pressures for compounds 2 (a, e) and 3 (a, e) are probably due to the hydrophobicity and polarity of the compounds. Amongst all the compounds, 2a is more hydrophobic due to the long alkyl chain and ester group. In contrast, compound 3e is more polarizable in nature due to the presence of a carboxylic acid (COOH) group and a small alkyl chain than others.

**Fig. 5:**
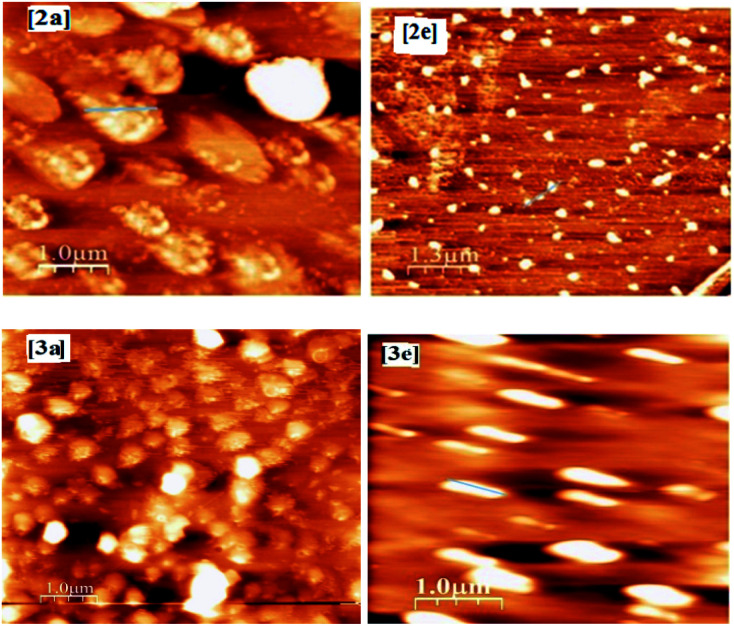
AFM images of spin-coated films for compounds 2a, 2e, 3a and 3e.

In order to have a deep idea about the surface morphologies of the thin films, we investigated the atomic force microscopic (AFM) images of spin-coated films. Fig. 5 shows the AFM images of compounds 2a, 2e, 3a and 3e in thin films deposited onto the smooth silicon substrate. The AFM images of spin-coated films revealed the sphere-like structures of aggregated 2a, 2e, 3a and 3e in the films, but the AFM line spectra of films of 2a and 2e indicated the clustering nature of the aggregates (see ESI†). Interestingly, all the sphere-like aggregates are uniformly distributed throughout the films, albeit they exhibited a small variation in dimensions as well as in heights. Since the dimension of the individual molecules is beyond the scope of resolution, it is not possible to distinguish individual molecules. After the analysis of height profile and line spectra (see ESI†) of AFM images (with respect to the empty substrate), it is estimated that the dimensions of sphere-like particles for compounds 2a, 2e, 3a and 3e are 200–250 nm, 150–250 nm, 200–300 nm and 120–170 nm respectively and heights are 10–12 nm, 10–12 nm, 12–14 nm and 7–8 nm respectively. As a whole, AFM images give us visual information about the formation of defined nano-structure aggregates (spherical in shape) of the compounds in the films.

**Fig. 6:**
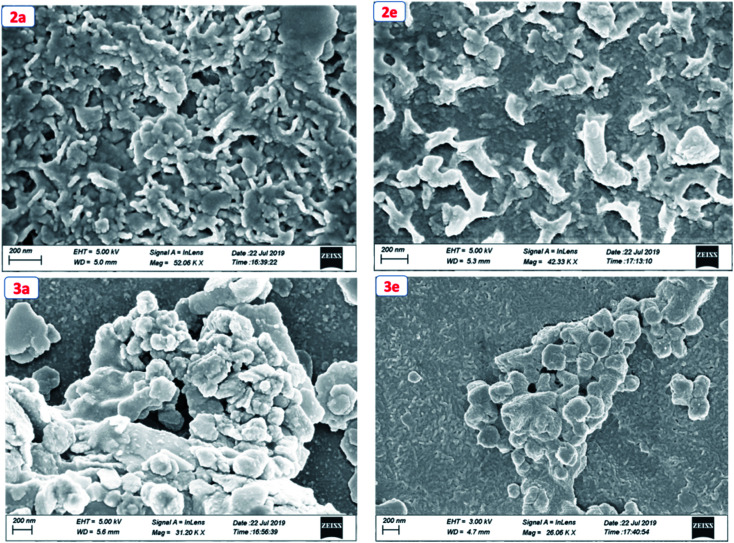
Scanning electron microscopic (SEM) images of compounds 2a, 2e, 3a and 3e.

To get the insight into the surface morphology of the films prepared by spin-coated techniques, scanning electron microscopic (SEM) studies were carried out for four selected compounds by taking two from each series. Fig. 6 shows the SEM images of the compounds 2a, 2e, 3a and 3e. These images reveal that the molecules of the compounds 2a and 2e aggregated to form a cluster-like morphology, whereas 3a and 3e show a sphere-type surface morphology. We anticipated that the possible additional interaction *via* H-bonding in 3a and 3e aggregated in such a way that they appeared as spherical in shape but in other series (2a and 2e) due to the presence of the ester group, they aggregated to form a cluster-like morphology.

### *I*–*V* characteristic studies of the compounds 2a–e and 3a–e

#### Switching behaviour

**Fig. 7:**
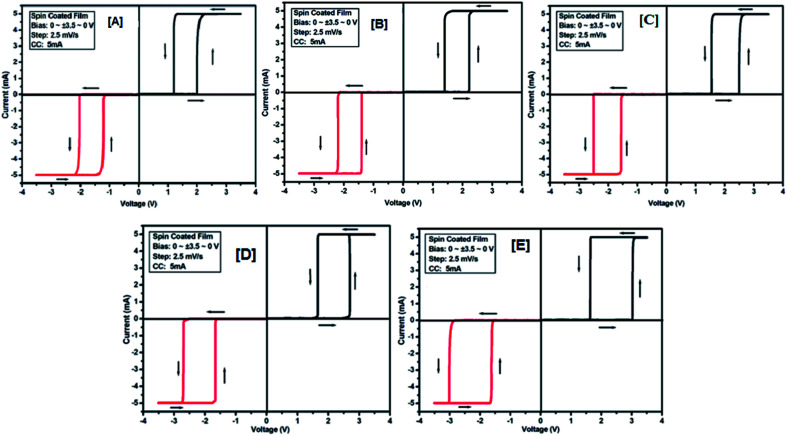
*I*–*V* characteristic for threshold switching of 2a [A], 2b [B], 2c [C], 2d [D] and 2e [E] for compounds 2a–e of the Au/2a–e/ITO device based on spin-cast films for two sweep direction. Initial sweep voltages (A–E) for all the devices were ±3.5 V at 5 mA compliance current. Arrows indicate the sweep direction of applied voltage.

**Fig. 8:**
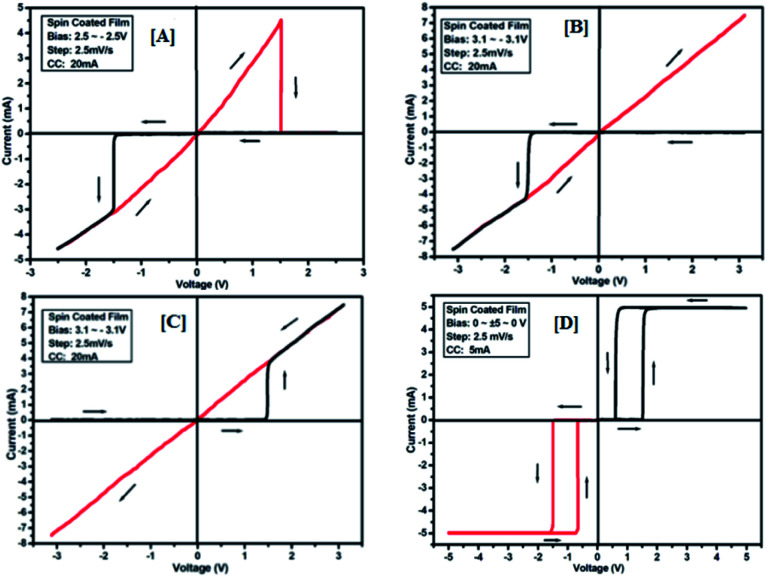
*I*–*V* characteristics for bipolar resistive switching (A, B and C) and threshold switching (D) for compound 3a of the Au/3a/ITO device based on spin-cast films for two sweep directions. Initial sweep voltages were (A) ±2.5 V, (B and C) ±3.1 V at 20 mA compliance current and (D) ±5 V at 5 mA compliance current. Arrows indicate the sweep direction of the applied voltage.

**Fig. 9:**
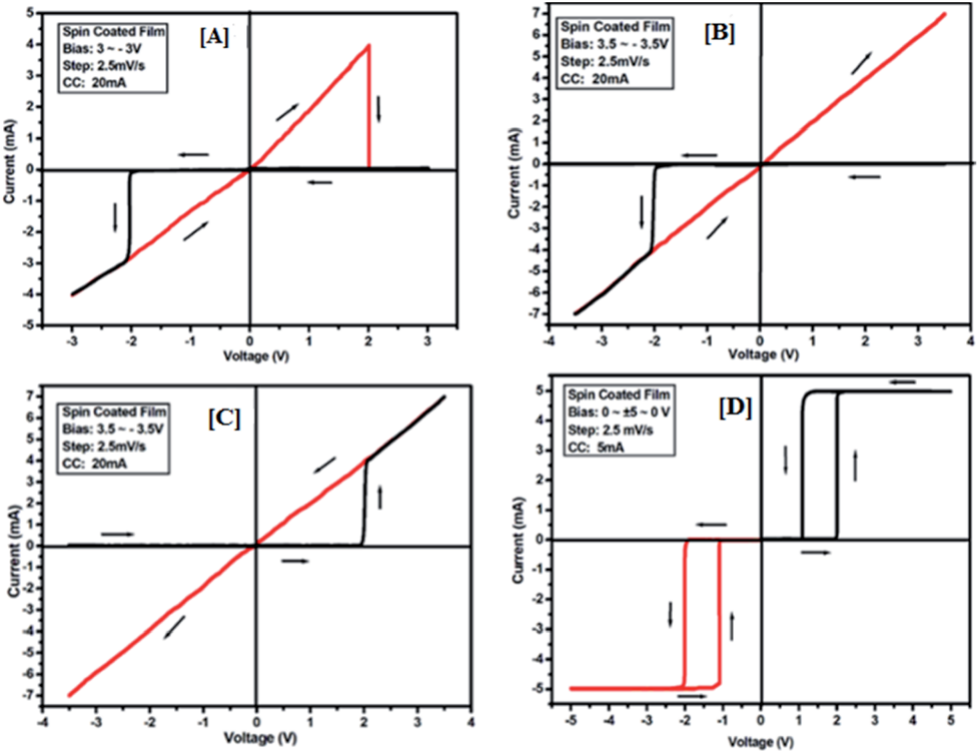
*I*–*V* characteristics for bipolar resistive switching (A, B and C) and threshold switching (D) for compound 3b of the Au/3b/ITO device based on spin-cast films for two sweep directions. Initial sweep voltages were (A) ±3 V, (B and C) ±3.5 V at 20 mA compliance current and (D) ±5 V at 5 mA compliance current. Arrows indicate the sweep direction of the applied voltage.

**Fig. 10:**
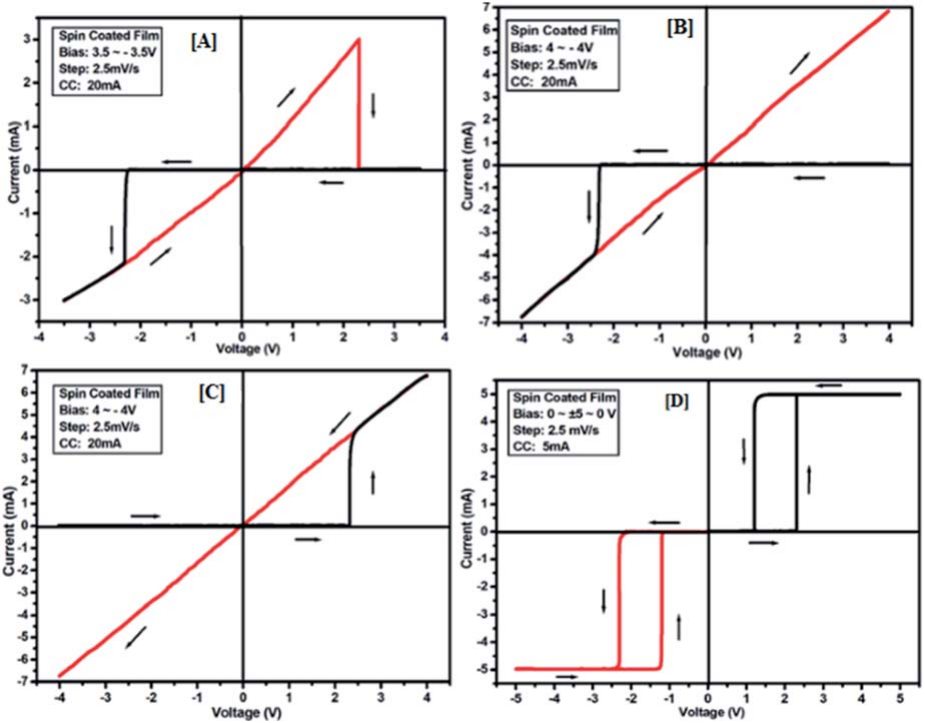
*I*–*V* characteristics for bipolar resistive switching (A, B and C) and threshold switching (D) for compound 3c of the Au/3c/ITO device based on spin-cast films for two sweep direction. Initial sweep voltages were (A) ±3.5 V, (B and C) ±4 V at 20 mA compliance current and (D) ±5 V at 5 mA compliance current. Arrows indicate the sweep direction of the applied voltage.

**Fig. 11:**
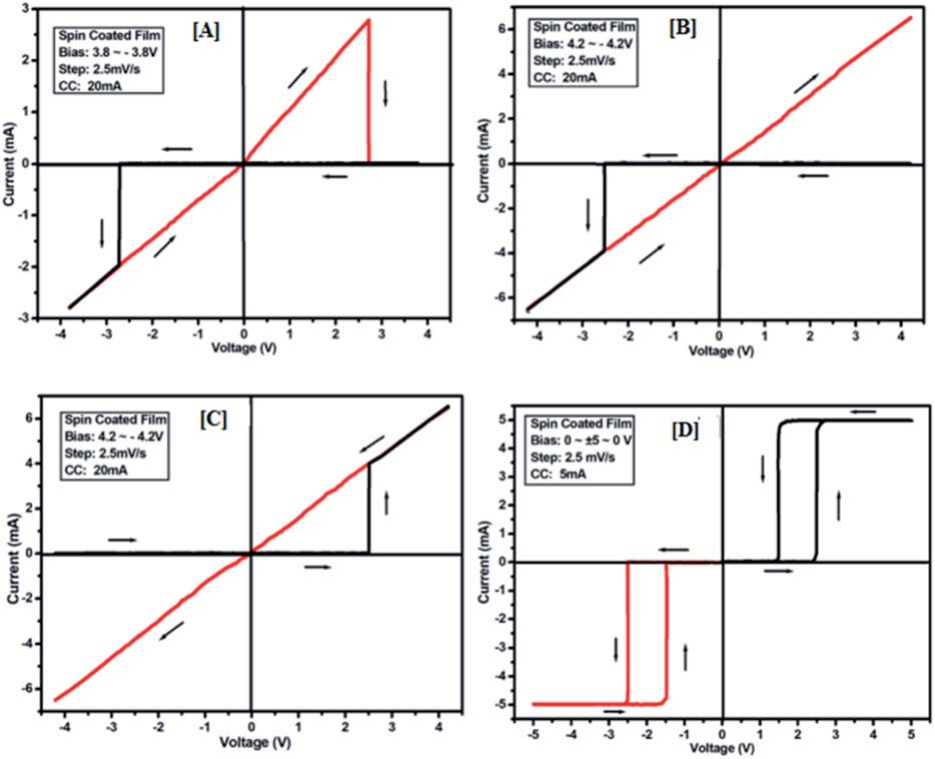
*I*–*V* characteristics for bipolar resistive switching (A, B and C) and threshold switching (D) for compound 3d of the Au/3d/ITO device based on spin-cast films for two sweep direction. Initial sweep voltages were (A) ±3.8 V, (B and C) ±4.2 V at 20 mA compliance current and (D) ±5 V at 5 mA compliance current. Arrows indicate the sweep direction of the applied voltage.

**Fig. 12:**
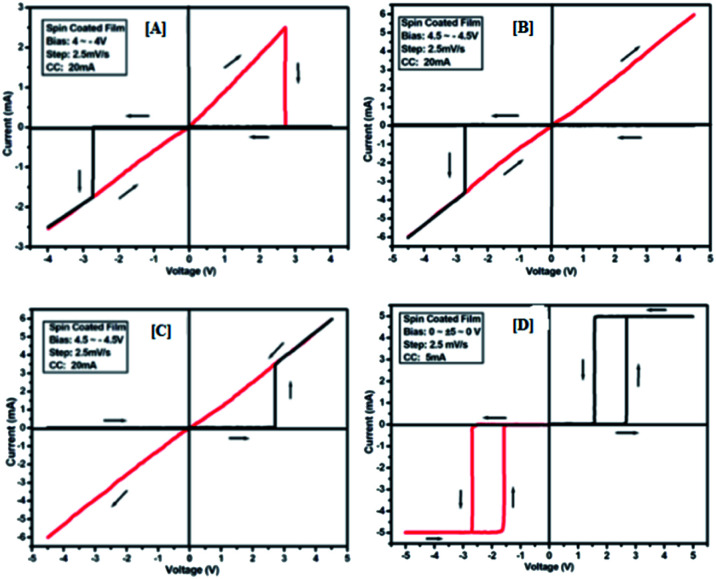
*I*–*V* characteristics for bipolar resistive switching (A, B and C) and threshold switching (D) for compound 3e of the Au/3e/ITO device based on spin-cast films for two sweep directions. Initial sweep voltages were (A) ± 4 V, (B, C) ± 4.5 V at 20 mA compliance current and (D) ± 5 V at 5 mA compliance current. Arrows indicate the sweep direction of the applied voltage.

On the basis of current–voltage (*I*–*V*) characteristics, there are two types of switching, namely, volatile and non-volatile switching. According to the volatility, there are two types of resistance switching: threshold and memory switching and based on the non-volatility, these are bipolar and unipolar switching. On the survey of the literature, it was found that, based on the polarity of the compounds and applied voltage, bipolar switching shows bidirectional resistance.^37,38^ At a certain voltage, non-volatile memory switching, switched on both the high-resistance and low-resistance states and at the low voltage, threshold switching shows only the high-resistance state.^39^ Nowadays, extensive research is carried out to exploit the electrical switching resistance (low and high) as a potential application in non-volatile memory devices for future generation.^40,41^ Electrically induced resistive memory switching and threshold switching phenomena in organic compounds are of great interest to develop novel electronic devices. It is possible to have different switching modes by adjusting the device configuration and measurement protocols. Moreover, a variety of mechanisms are responsible for the electrical switching behaviour (bipolar and threshold switching) of organic compounds, such as conformational changing, rotation of functional group, charge transfer, oxidation–reduction process, filamentary conduction, space charge and traps, and ionic conduction.^42–44^ From UV, fluorescence and morphological studies of the synthesized compounds 2a–e and 3a–e indicated that they formed H-aggregates, *i.e.* in films they associated by face-to-face assembly through hydrophobic–hydrophobic interactions between long alkyl chains and π–π stacking between aromatic cores. These aggregation behaviours prompt us to conduct the electrical switching behaviour of compounds 2a–e and 3a–e assembled onto spin-coated films. In this purpose, by the variation of compounds (2a–e and 3a–e), we designed various (ten types) switching devices: (i) Au/2x/ITO (where x = a, b, c, d and e) and (ii) Au/3y/ITO (where y = a, b, c, d and e) and studied their resistive bipolar memory switching and threshold switching behaviour by adjusting the bias voltage, compliance current, sweep direction, *etc.*^45^ The configuration of the devices and details of initial sweep voltages and compliances current are shown in Fig. 7–12.

### *I*–*V* characteristic of molecules (2a–e) in spin-coated films

To study the electrical switching behaviour of coumarin derivatives 2a–e, the device having configurations Au/2x/ITO (where x = a, b, c, d and e) was prepared by spreading 1–2 drops of solutions on an ITO-coated glass substrate spun at a rotating speed of 1800 rpm. The resulting spin-coated films are dried sufficiently in vacuum at room temperature before the computation of *I*–*V* characteristics. The current–voltage behaviour was recorded by applying the voltage tuning from +*V*_max_ to −*V*_max_ and *vice versa*. The voltage was imposed on the top electrode (gold tip), whereas the bottom electrode (ITO-coated glass substrate) was held on ground potential.

The electrical (*I*–*V*) measurement was performed by giving an active area of 1 mm^2^ for measurement. Same/different scanning/tuning voltages were applied to measure the *I*–*V* curves for all the devices at a voltage scan rate of 2.5 mV s^−1^. Fig. 7[A]–[E] shows the *I*–*V* curves of 2a–e. The device Au/2a/ITO exhibits a low conducting state (OFF state, 15.42 × 10^4^ Ω) for tuning the voltage range from +3.5 V to −3.5 V (scanning range was 0 to +3.5 to 0 to −3.5 to 0) (Fig. 7[A]), but the current was increased when the voltage approaches the threshold voltage (about 2 V), showing its high conducting state (ON state, 138.72 × 10^1^ Ω). A similar procedure was followed for all other devices: Au/2b/ITO, Au/2c/ITO and Au/2d/ITO. The device Au/2e/ITO exhibits a threshold voltage of 3.0, and the resistance recorded in the OFF state is 5.11 × 10^4^ Ω and the on state is 165.49 × 10^1^ Ω respectively. The results of *I*–*V* characteristics of these devices are listed in Table 2. From the results tabulated in Table 2, it is clear that with the decrease in the alkoxy chain length of 2 in the devices Au/2x/ITO, the threshold voltages increase with the decrease in resistance in the OFF state, whereas in the ON state, the resistance increases. No bipolar switching was observed in this series probably due to the hydrophobic and non-polar nature of 2a–e.

**Table 2:** Different parameters of *I*–*V* characteristics for Au/2a–e/ITO devices

Device configuration	Threshold switching			Resistances at	
	Threshold voltage (V)	Maximum compliance current (mA)	Minimum compliance current (mA)	OFF state (Ω)	ON state (Ω)
Au/(2a)/ITO	2	10	2	15.42 × 10^4^	138.72 × 10^1^
Au/(2b)/ITO	2.2	10	2	12.79 × 10^4^	146.68 × 10^1^
Au/(2c)/ITO	2.5	10	2	10.01 × 10^4^	150.93 × 10^1^
Au/(2d)/ITO	2.7	10	2	8.21 × 10^4^	161.92 × 10^1^
Au/(2e)/ITO	3	10	2	5.11 × 10^4^	165.49 × 10^1^

### *I*–*V* characteristic of molecules (3a–e) in spin-coated films

To study the switching behaviour of the compounds 3a–e, a device having configurations Au/3y/ITO (where y = a, b, c, d and e) was prepared by spin-coating techniques. The current–voltage behaviour measurement was studied by tuning the voltage from +*V*_max_ to −*V*_max_ and *vice versa* (with an increment of 0.1 V for *V*_max_ each time). The *I*–*V* computation curve of the device having the configuration Au/3a/ITO for tuning the voltage range from +2.5 V to −2.5 V is shown in Fig. 8[A]. Initially, the above-mentioned device exhibits a low conducting state, by tuning the voltage range from +*V*_max_ to −*V*_max_, but when the voltage closes to a threshold value of 1.5 V, it shows a high conducting state, and during scanning reverse *i.e.* −*V*_max_ to +*V*_max_, the device shows transition from a high conducting state to a low conducting state (ON to OFF state) after reaching the threshold voltage (Fig. 8[A]).

The similar bipolar switch could be formed independently by a maximum tuning voltage up to 3 V. It was found that the device sweeps to its ON state during tuning the voltage from +*V*_max_ to −*V*_max_ when the voltage exceeds 3 V, and during tuning −*V*_max_ to +*V*_max_ it does not return back to its low conducting state (Fig. 8[B]). Similarly, it was also observed that, when the voltage exceeds 3 V, then driving the device to its high conducting state (scanning from −*V*_max_ to +*V*_max_) does not return back to its low conducting state (Fig. 8[C]) during scanning from +*V*_max_ to −*V*_max_. It was noticed that by tuning, the compliance current transition from memory switching to threshold switching becomes irreversible. The bipolar switching cannot be recovered if the threshold switching is completely formed. Moreover, when applied to a higher voltage, *i.e.* 3.1 V in the device, it gets short circuit and shows only one way switching, it may be due to the organic layer between the electrodes in the ON state and the OFF state.^46^ However, in threshold and bipolar switching devices, the current in the OFF state is due to the low leakage of current.

The results of the ON state and OFF state resistances (Ω) calculated from the *I*–*V* curve are listed in Table 3. Adjusting the measurement and all other protocols we have also investigated the threshold switching from the observed bipolar resistive switching of the same device. In case of both the forward and the reverse bias, the device shows threshold switching at fixed compliance current (5 mA). For threshold switching having the same configuration of the device (Au/3a/ITO) with scanning range 0 ± 5 to 0 V (Fig. 8[D]) showed a similar type of threshold switching by lowering the compliance current up to 2 mA. It was observed that there is no effect on the threshold switching if the sweep voltage range is more than 3.1 V. It is because the current increases besides the compliance level, which has no effect by increasing the sweep voltage and the device do not get short-circuited. In all the devices, the active junction area was 1 mm^2^ for *I*–*V* measurement. Similar trends were also observed for other devices Au/3b/ITO, Au/3c/ITO, Au/3d/ITO and Au/3e/ITO (Fig. 9–12). The results of all the devices are listed in Table 3.

**Table 3:** Different parameters of *I*–*V* characteristics for Au/3a–e/ITO devices

Device configuration	Bipolar switching			Threshold switching			Resistances at	
	Threshold voltage (V)	Maximum allowed scanning voltage (V)	Shorted scanning voltage (V)	Threshold voltage (V)	Maximum compliance current (mA)	Minimum compliance current (mA)	OFF state (Ω)	ON state (Ω)
Au/(3a)/ITO	1.5	2.5	3.1	1.5	5	2	2.7 × 10^4^	43.00 × 10^1^
Au/(3b)/ITO	2	3	3.5	2	5	2	3.3 × 10^4^	51.30 × 10^1^
Au/(3c)/ITO	2.3	3.5	4	2.3	5	2	4.3 × 10^4^	79.20 × 10^1^
Au/(3d)/ITO	2.5	3.8	4.2	2.5	5	2	5.8 × 10^4^	101.7 × 10^1^
Au/(3e)/ITO	2.7	4	4.5	2.7	5	2	6.7 × 10^4^	104.4 × 10^1^

During the *I*–*V* measurement, a gold tip, softly touching the surface of the prepared thin films (spin-coated), was used as the top electrode for an active junction area of 1 mm^2^ for measurement. However, we also measured the *I*–*V* characteristics for different devices by varying the active junction area, but no significant variation is observed.

From the study of the current–voltage measurement, it was found that the synthesised coumarin derivatives 2a–e show threshold switching, but compounds 3a–e show threshold as well as bipolar resistive switching. The observed bipolar switching behaviour may be explained due to polarizable carboxylic acid groups (–COOH) and electron-donating long alkoxy groups (–OC_18_H_37_, –OC_16_H_33_, –OC_14_H_29_, –OC_12_H_25_, –OC_10_H_21_) at position 3 of the coumarin ring. When the devices of coumarin derivatives 3a–e were scanned from a positive voltage to a negative voltage, it shows a very low OFF state leakage current. It may be due to the attractions between the presence of π-electron clouds in the cyclic ring of the coumarin core and the electron acceptor carboxylic acid group. The leakage of current may be restored by giving the electrons in the system by an electron-donating alkoxy group. Due to the presence of delocalized π-electrons in the entire molecules, it can be responsible to bear a conduction pathway to result in a high-conducting ON state. Moreover, when extra electrons were added through an electron-withdrawing alkyl group, it can oxidise the compound and the device returns to its OFF state during scanning from −*V*_max_ to +*V*_max_. In case of threshold switching, the difference in work functions between the metals used as electrodes and the resulting built-in internal field determine the bias direction as well as at which the electron injection to the organic layer is favourable. Therefore, depending on the bias, the conjugation in the backbone of the molecule is presumably extended and this results in a high-conducting state. Moreover, due to the presence of the –COOH group, organizational behaviours of 3a–e are considerably different from those of 2a–e, as the hydrogen bond donor–acceptor phenomenon makes a highly organized assembly for 3a–e in the film, which is also reflected in the SEM images. However, it cannot be ruled out that the coumarin molecule may undergo ring-opening due to the presence of water molecules around the device followed by oxidation of phenolic compounds so generated to quinone.^47^ Therefore, as the biases applied are very large, the possibility of such quinone–hydroquinone oxidation–reduction process pathways could be another acceptable mechanism in the present studies.

The carrier conduction involves both injection and transport. Hence, electro-reduction of 2a–e and 3a–e compounds occurred and the device switches to its high state at a forward bias. *I*–*V* characteristic for bipolar resistive switching and threshold switching for two sweep direction of the spin coated film based Au/2a–e/ITO and Au/3a–e/ITO devices was prepared by spin coating technique. Spin-coated films are quite thick and they have good control in assembling of molecules onto thin films. It was observed that the device get short circuited when the scanning voltage goes beyond certain maximum limiting values. During the ON state when the current passes through the devices, the organic layer in the device is heated up. With the increase in current due to the increase in bias voltage, the extent of heating also increases and the device gets damage. To prevent such damage, the current flowing in the memory devices should be kept below compliance.

## Conclusions

In summary, we have successfully explored novel coumarin derivatives appended with a long alkoxy chain on the seventh position of a coumarin-3-carboxylate/carboxylic acid core as potential candidates for futuristic materials to make devices of specific interest. The synthesised materials were characterized by different analytical tools such as NMR and mass spectroscopy, UV, fluorescence, AFM and SEM studies as well as *I*–*V* characteristic mapping respectively. From the *I*–*V* characteristics, it was found that at room temperature, the spin-coated films of coumarin derivatives containing an ester functional group exhibited a threshold switching behaviour, whereas the derivatives containing a carboxylic acid functional group shows both threshold and bipolar switching behaviours. We also noticed that the *I*–*V* characteristic features of the synthesized materials depend on the length of the alkyl chain of individual series. *I*–*V* characterized data reveal that these coumarin derivatives, particularly coumarin carboxylic acids 3a–e, could be useful as innovative futuristic materials for organic electronics and valuable applications in chemical, material and medical sciences.

## Author contributions

S. M. designed the work, A. R. P. synthesised the materials, NMR and mass spectral analyses, A. R. P., B. D. and S. S. performed all experiments and data analysis with input from S. A. H. and S. M., A. R. P and B. D. wrote the manuscript.

## Conflicts of interest

There are no conflicts to declare.

## Supplementary Material

RA-011-D1RA00762A-s001

## References

[cit1] Kang J., Sangwan V. K., Wood J. D., Hersam M. C. (2017). Acc. Chem. Res..

[cit2] Gong C., Hu K., Wang X., Wangyang P., Yan C., Chu J., Liao M., Dai L., Zhai T., Wang C., Li L., Xiong J. (2018). Adv. Funct. Mater..

[cit3] Klauk H. (2010). Chem. Soc. Rev..

[cit4] Wu Q.-H., Zhao P., Chen G. (2015). Org. Electron..

[cit5] Ni W., Li M., Kan B., Zuo Y., Zhang Q., Long G., Feng H., Wan X., Chen Y. (2014). Org. Electron..

[cit6] Chen G., Si C., Zhang P., Wei B., Zhang J., Hong Z., Sasabe H., Kido J. (2017). Org. Electron..

[cit7] Guerrero A., Pfannmoller M., Kovalenko A., Ripolles T. S., Heidari H., Bals S., Kaufmann L.-D., Bisquert J., Garcia-Belmonte G. (2015). Org. Electron..

[cit8] Lai Y.-C., Hsu F.-C., Chen J.-Y., He J.-H., Chang T.-C., Hsieh Y.-P., Lin T. Y., Yang Y.-J., Chen Y.-F. (2013). Adv. Mater..

[cit9] Sun X.-Y., Liu T., Sun J., Wang X.-J. (2020). RSC Adv..

[cit10] Bhattacharya S. S., Paul S. K., Mandal A., Banerjee N., Boujedaini A. R. (2009). Eur. J. Pharmacol..

[cit11] Ito C., Itoigawa M., Onoda S., Hosokawa A., Ruangrungsi N., Okuda T., Tokuda H., Nishino H., Furukawa H. (2005). Phytochemistry.

[cit12] Gudasi K. B., Patil M. S., Vadavi R. S. (2008). Eur. J. Med. Chem..

[cit13] Sardori S., Mori Y., Micetich R. G., Nishibe S., Daneshtalab M. (1999). Bioorg. Med. Chem..

[cit14] Gacche R. N., Gond D. S., Dhole N. A., Dawane B. S. (2006). J. Enzyme Inhib. Med. Chem..

[cit15] Manolov I., Maichle-Moessmer C., Danchev N. (2006). Eur. J. Med. Chem..

[cit16] Ivana K., Andrea J., Pavol K. (2006). Beilstein J. Org. Chem..

[cit17] Lee K. S., Kim H. J., Kim G. H., Shin I., Hong J. I. (2008). Org. Lett..

[cit18] Zhou S. H., Jia J. H., Gao J. R., Han L., Li Y. J., Sheng W. J. (2010). Dyes Pigm..

[cit19] Harishkumar H. N., Mahadevanand K. M., Masagalli J. N. (2012). S. Afr. J. Chem..

[cit20] Murata C., Masuda T., Kamochi Y., Todoroki K., Yoshida H., Nohta H., Yamaguchi M., Takadate A. (2005). Chem. Pharm. Bull..

[cit21] Kuo P. Y., Yang D. Y. (2008). J. Org. Chem..

[cit22] Körner P. O., Shallcross R. C., Maibach E., Köhnen A., Meerholz K. (2014). Org. Electron..

[cit23] Yam V. W. W., Song H. O., Chan S. T. W., Zhu N., Tao C. H., Wong K. M. C., Wu L. X. (2009). J. Phys. Chem. C.

[cit24] Cui S. L., Lin X. F., Wang Y. G. (2006). Org. Lett..

[cit25] Lee K. S., Kim H. J., Kim G. H., Shin I., Hong J. I. (2008). Org. Lett..

[cit26] Barooah N., Sundararajan M., Mohanty J., Bhasikuttan A. C. (2014). J. Phys. Chem. B.

[cit27] Li J., Zhang C. F., Yang S. H., Yang W. C., Yang G. F. (2014). Anal. Chem..

[cit28] Trenor S. R., Shultz A. R., Love B. J., Long T. E. (2004). Chem. Rev..

[cit29] Yi C. L., Sun J. H., Zhao D. H., Hu Q., Liu X. Y., Jiang M. (2014). Langmuir.

[cit30] Wu H. Q., Zhao P., Chen G. (2015). Org. Electron..

[cit31] Cui S. L., Lin X. F., Wang Y. G. (2006). Org. Lett..

[cit32] Ren X., Kondakova M. E., Giesen D. J., Rajeswaran M., Madaras M., Lenhart W. C. (2010). Inorg. Chem..

[cit33] Koo J. R., Lee H. S., Ha Y., Choi Y. H., Kim Y. K. (2003). Thin Solid Films.

[cit34] Gao S., Yi X., Shang J., Liu G., Li R.-W. (2019). Chem. Soc. Rev..

[cit35] Bigi F., Chesini L., Maggi R., Sartori G. (1999). J. Org. Chem..

[cit36] Ulman A. , An introduction to ultrathin organic films: from Langmuir-Blodgett to self-assembly, Academic Press, Boston, 1991

[cit37] Wu S. X., Xu L. M., Xing X. J., Chen S. M., Yuan Y. B., Liu Y. J., Yu Y. P., Li X. Y., Li S. W. (2008). Appl. Phys. Lett..

[cit38] Waser R., Dittmann R., Staikov G., Szot K. (2009). Adv. Mater..

[cit39] Chang S. H., Lee J. S., Chae S. C., Lee S. B., Liu C., Kahng B., Kim D. W., Noh T. W. (2009). Phys. Rev. Lett..

[cit40] Strukov D. B., Snider G. S., Stewart D. R., Williams R. S. (2008). Nature.

[cit41] Lee M. J., Lee C. B., Lee D., Lee S. R., Chang M., Hur J. H., Kim Y. B., Kim C. J., Seo D. H., Seo S., Chung U. I., Yoo I. K., Kim K. (2011). Nat. Mater..

[cit42] Sawa A. (2008). Mater. Today.

[cit43] Lee M.-J., Park Y., Suh D.-S., Lee E.-H., Seo S., Kim D.-C., Jung R., Kang B.-S., Ahn S. E., Lee C. B., Seo D. H., Cha Y.-K., Yoo I.-K., Kim J.-S., Park B. H. (2007). Adv. Mater..

[cit44] Hwang I., Lee M. J., Buh G. H., Bae J., Choi J., Kim J. S., Hong S., Kim Y. S., Byun I. S., Lee S. W., Ahn S. E., Kang B. S., Kang S. O., Park B. H. (2010). Appl. Phys. Lett..

[cit45] Peng H. Y., Li Y. F., Lin W. N., Wang Y. Z., Gao X. Y., Wu T. (2012). Sci. Rep..

[cit46] Siebeneicher P., Kleemann H., Leo K., Lüssem B. (2006). Appl. Phys. Lett..

[cit47] Ciampi S., James M., LeSaux G., Gaus K., Gooding J. J. (2012). J. Am. Chem. Soc..

