# Supplementary material for: 7-Alkoxy-appended coumarin derivatives: synthesis, photo-physical properties, aggregation behaviours and current–voltage (I–V) characteristic studies on thin films
Source: RSC Adv. 2021 Mar 10;11(17):10212–23. doi: 10.1039/d1ra00762a (PMC8695653; doi:10.1039/d1ra00762a)
Supplement: RA-011-D1RA00762A-s001 [file RA-011-D1RA00762A-s001.pdf]

Electronic Supplementary Material (ESI) for RSC Advances.
This journal is © The Royal Society of Chemistry 2021

7-Alkoxy appended coumarin derivatives: Synthesis, photo-physical properties, aggregation behaviours and current–voltage (I-V) characteristic studies on thin films

Abhijit Rudra Paul, Bapi Dey, Sudip Suklabaidya, Syed Arshad Hussain and Swapan Majumdar

[a] *Department of Chemistry, Tripura University, Suryamaninagar, 799 022, INDIA*
[b] *Department of Physics, Tripura University, Suryamaninagar, 799 022, INDIA*

Supporting information

Contents: Page

1. ^{1}H and ^{13}C spectra of **1** (Figure 1-2) 2
2. ^{1}H and ^{13}C spectra of **2a-e** (Figure 3-12) 3-7
3. ^{1}H and ^{13}C spectra of **3a-e (**Figure 13-22) 8-12
4. Fluorescence spectra of **2a-e** and **3a-e** in solution and film (Figure 23) 13-14
5. AFM images of thin film of **2a**, **2e**, **3a** and **3e** (Figure 24) 14

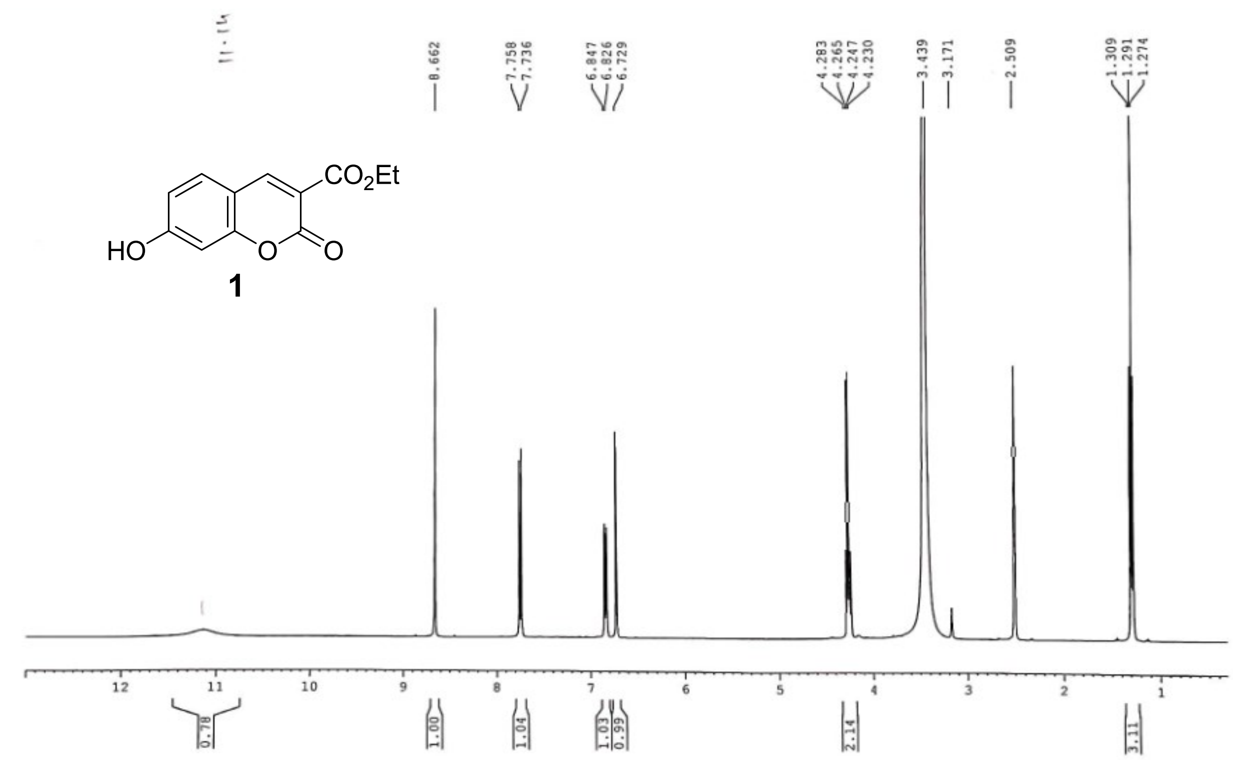

Figure 1: **^{1}H NMR spectrum of 1(400 MHz, DMSO-d$_6$)**

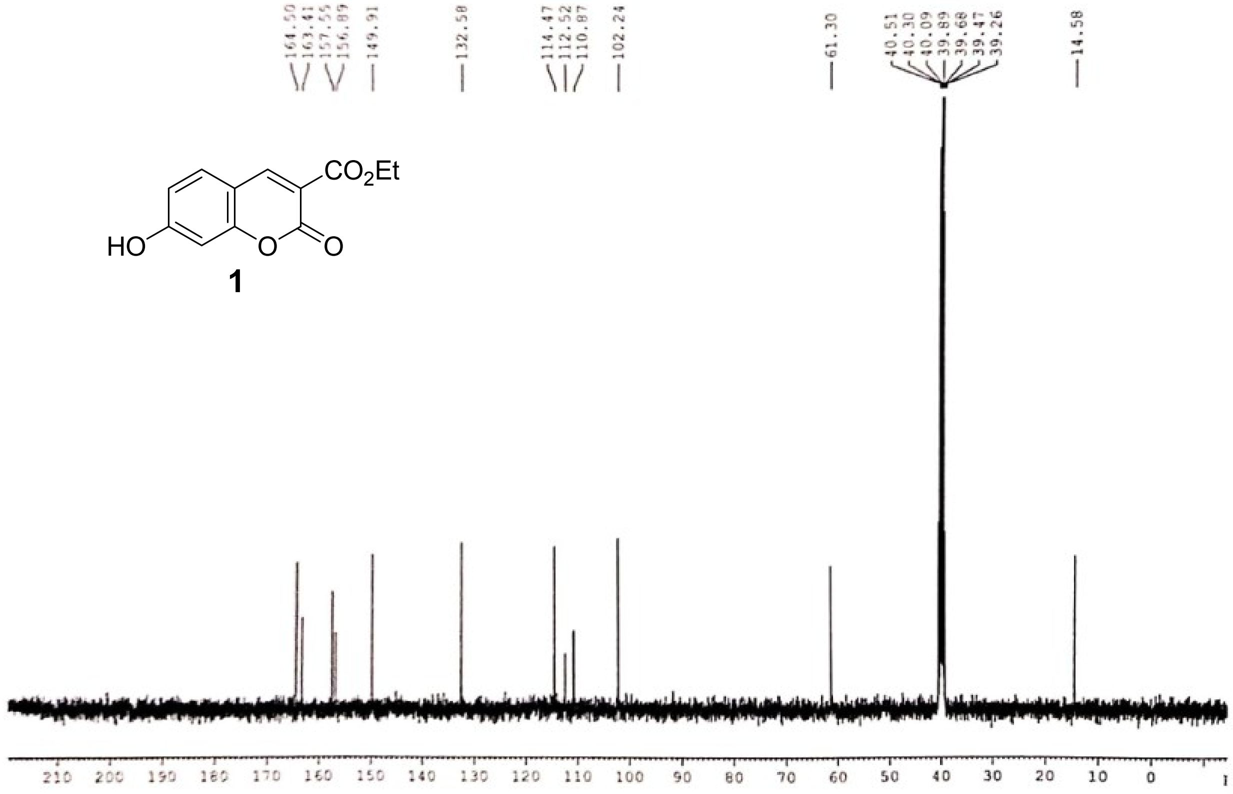

Figure 2: **^{13} C NMR spectrum of 1(100 MHz, DMSO-d$_6$)**

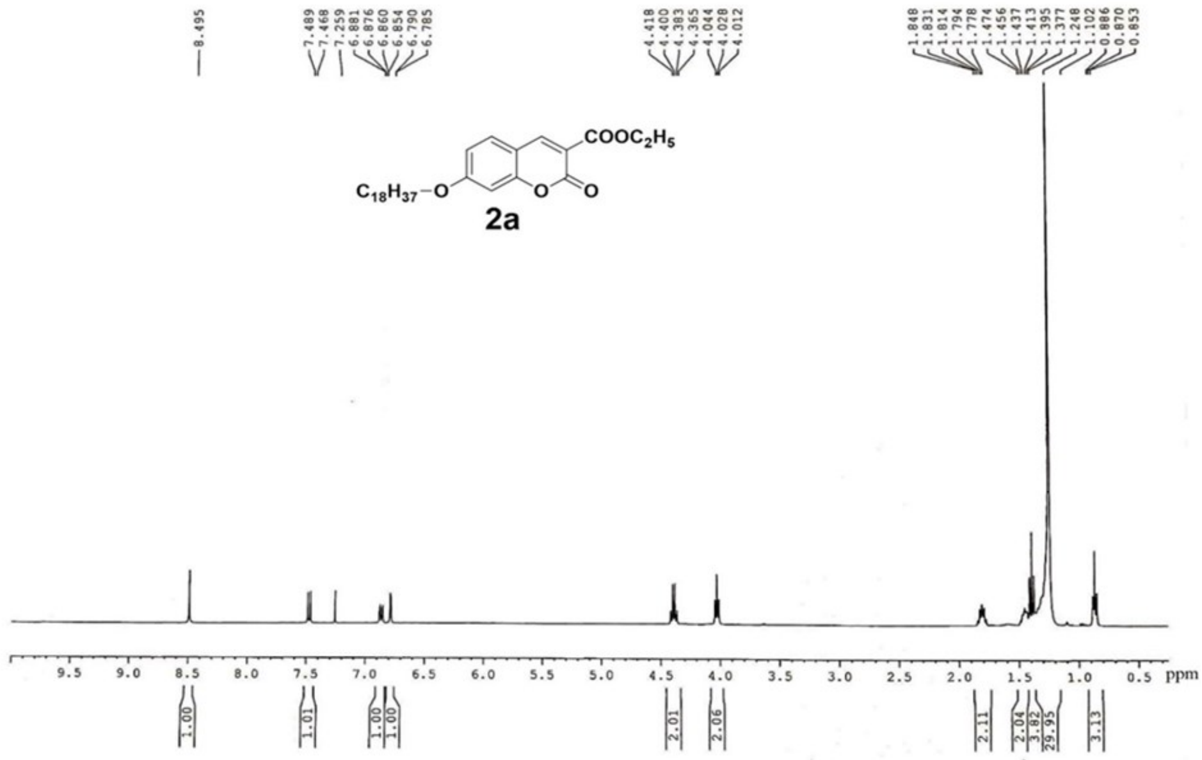

Figure 3: **^{1}H NMR spectrum of 2a (400 MHz, $CDCl_3$)**

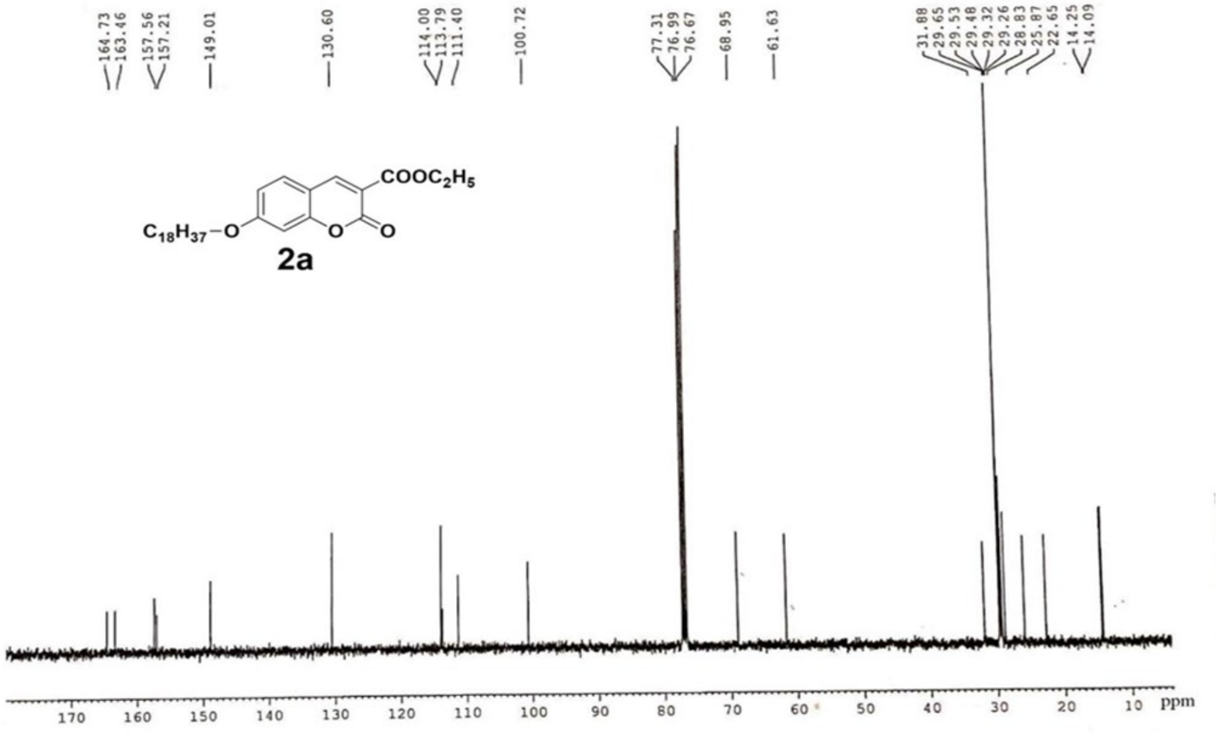

Figure 4: **^{13}C NMR spectrum of 2a (100 MHz, $CDCl_3$)**

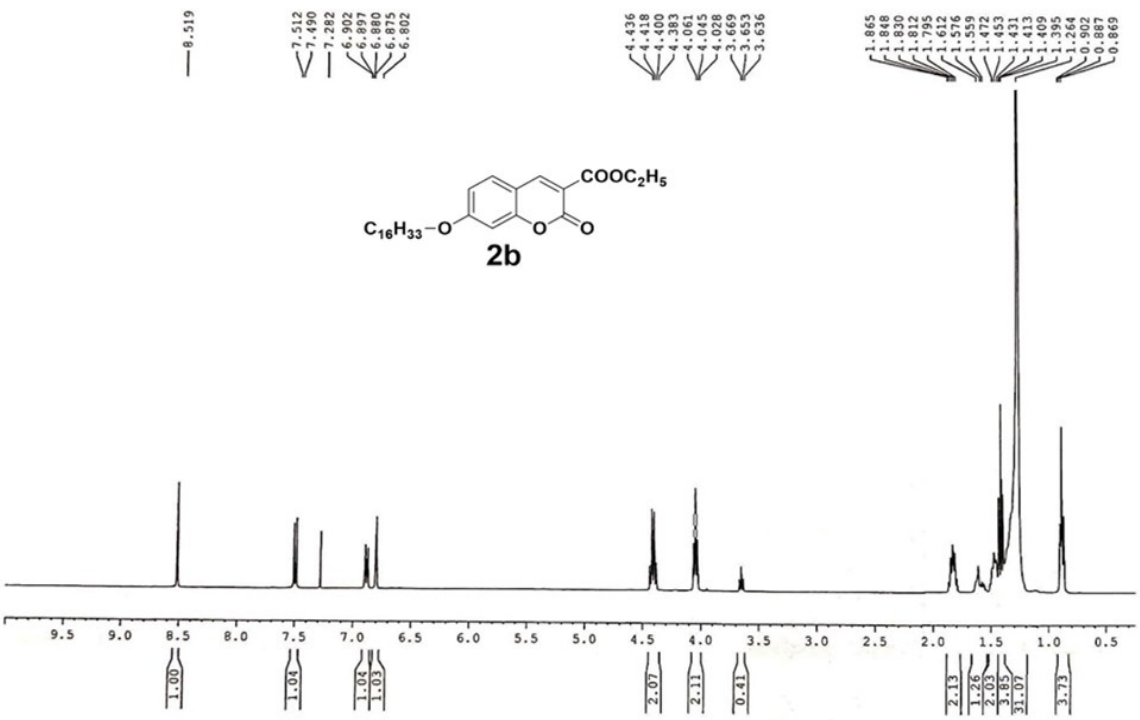

Figure 5: **^{1}H NMR spectrum of 2b (400 MHz, $CDCl_3$)**

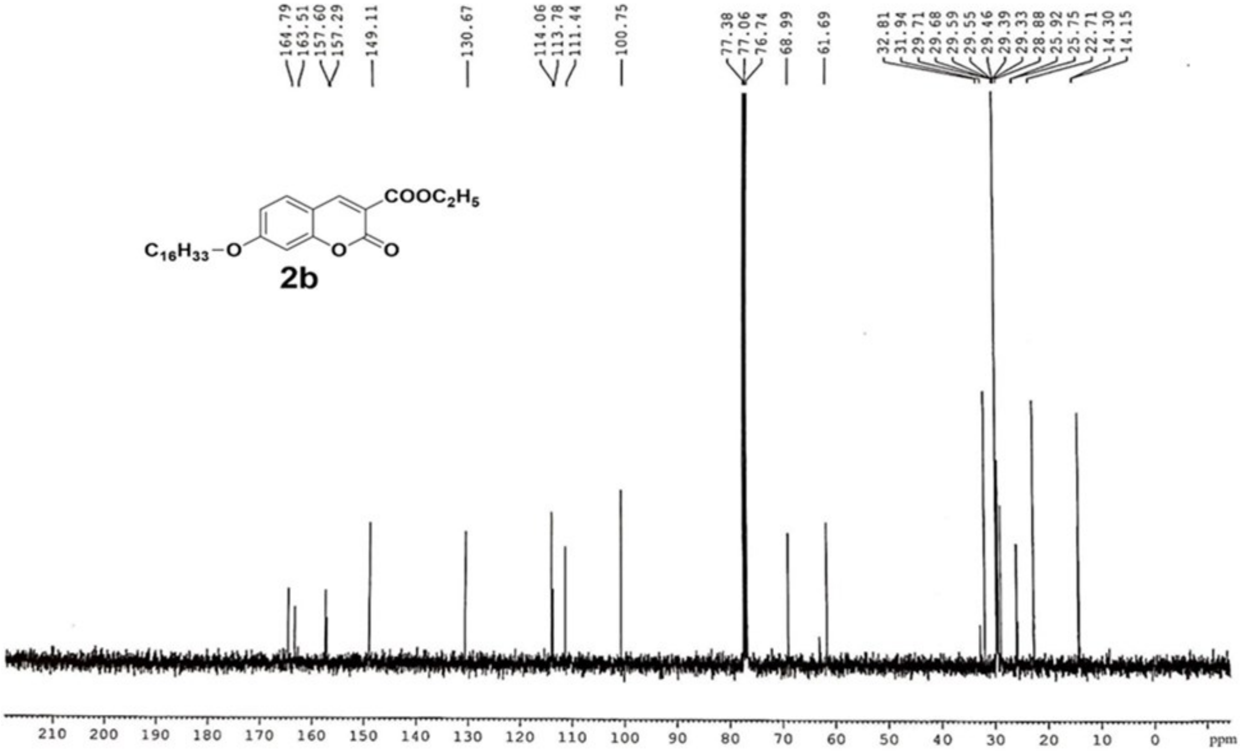

Figure 6: **^{13}C NMR spectrum of 2b (100 MHz, $CDCl_3$)**

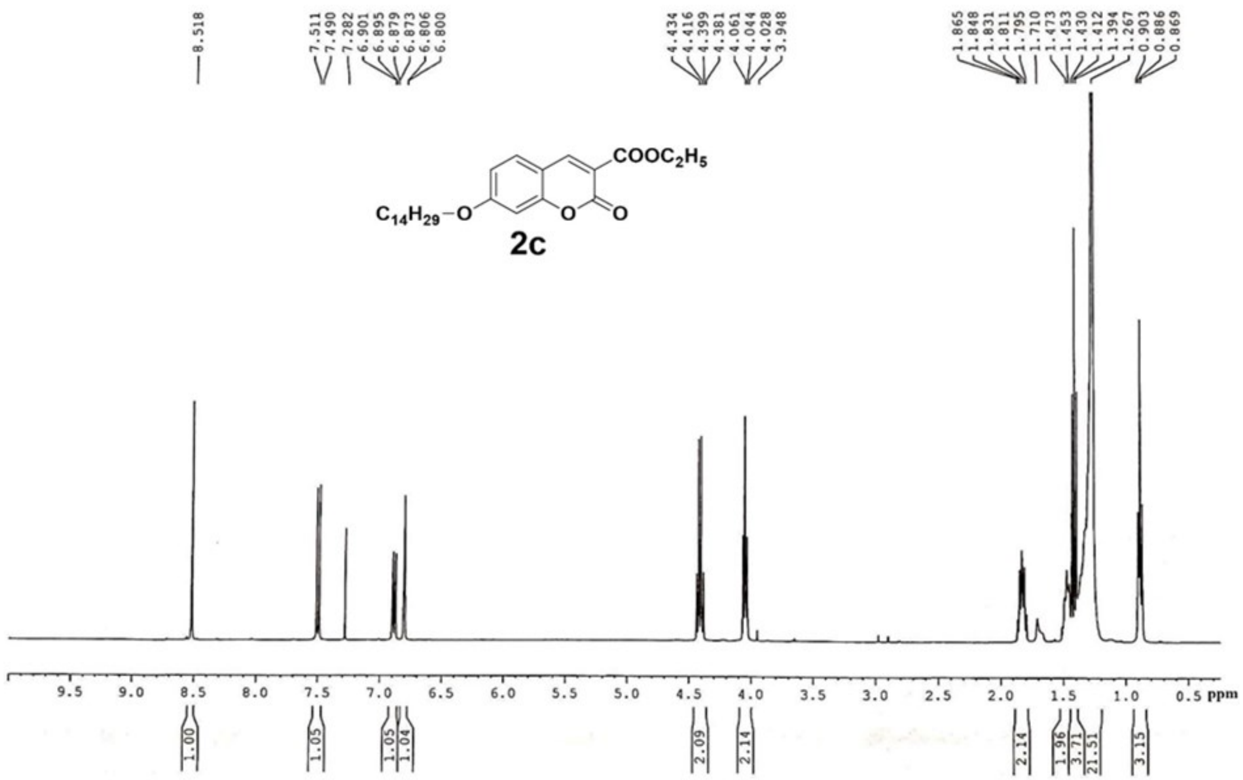

Figure 7: **^{1}H NMR spectrum of 2c (400 MHz, $CDCl_3$)**

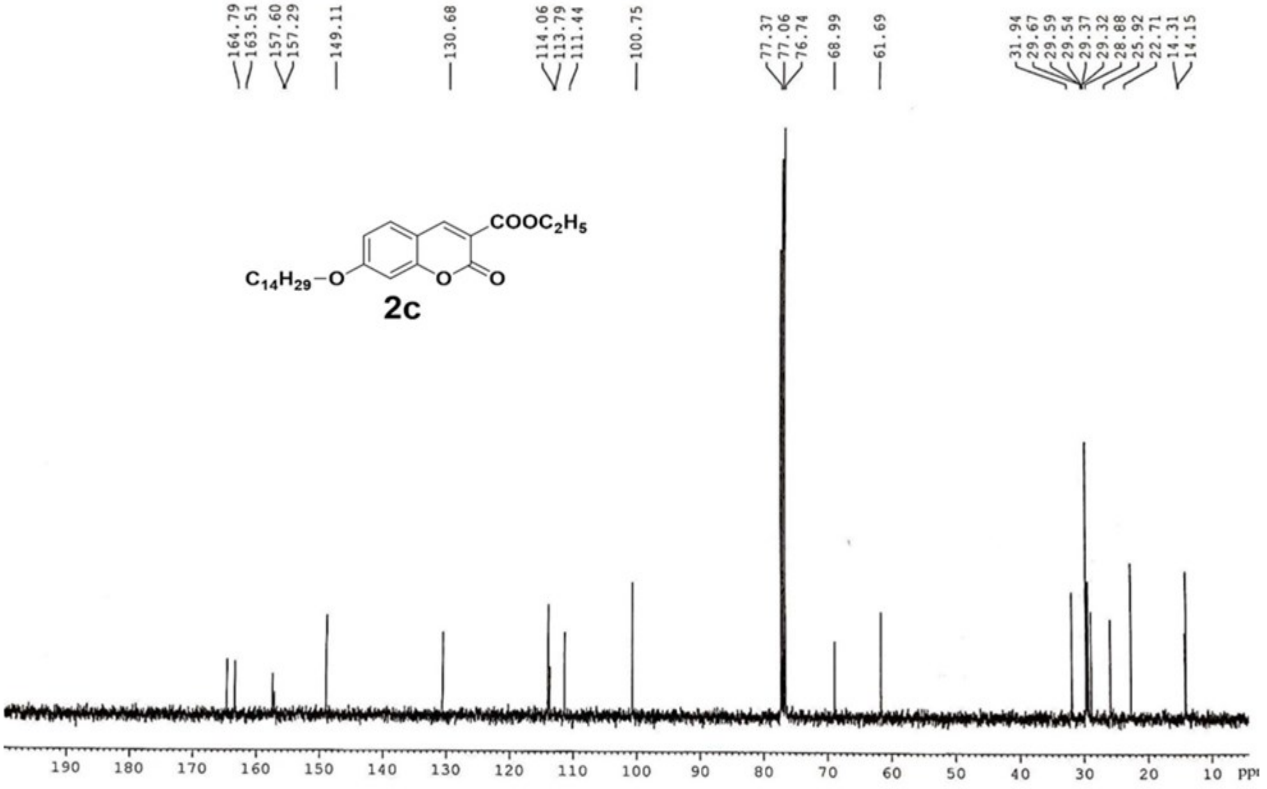

Figure 8: **^{13}C NMR spectrum of 2c (100 MHz, $CDCl_3$)**

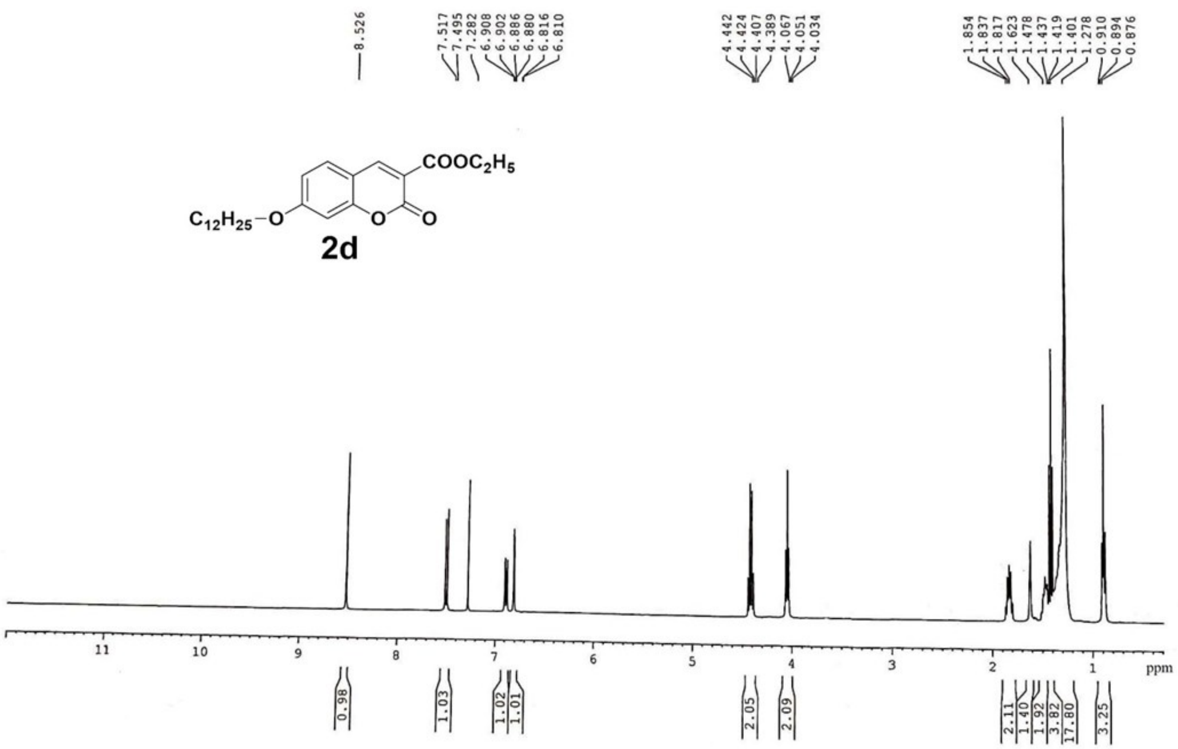

Figure 9: **^{1}H NMR spectrum of 2d (400 MHz, $CDCl_3$)**

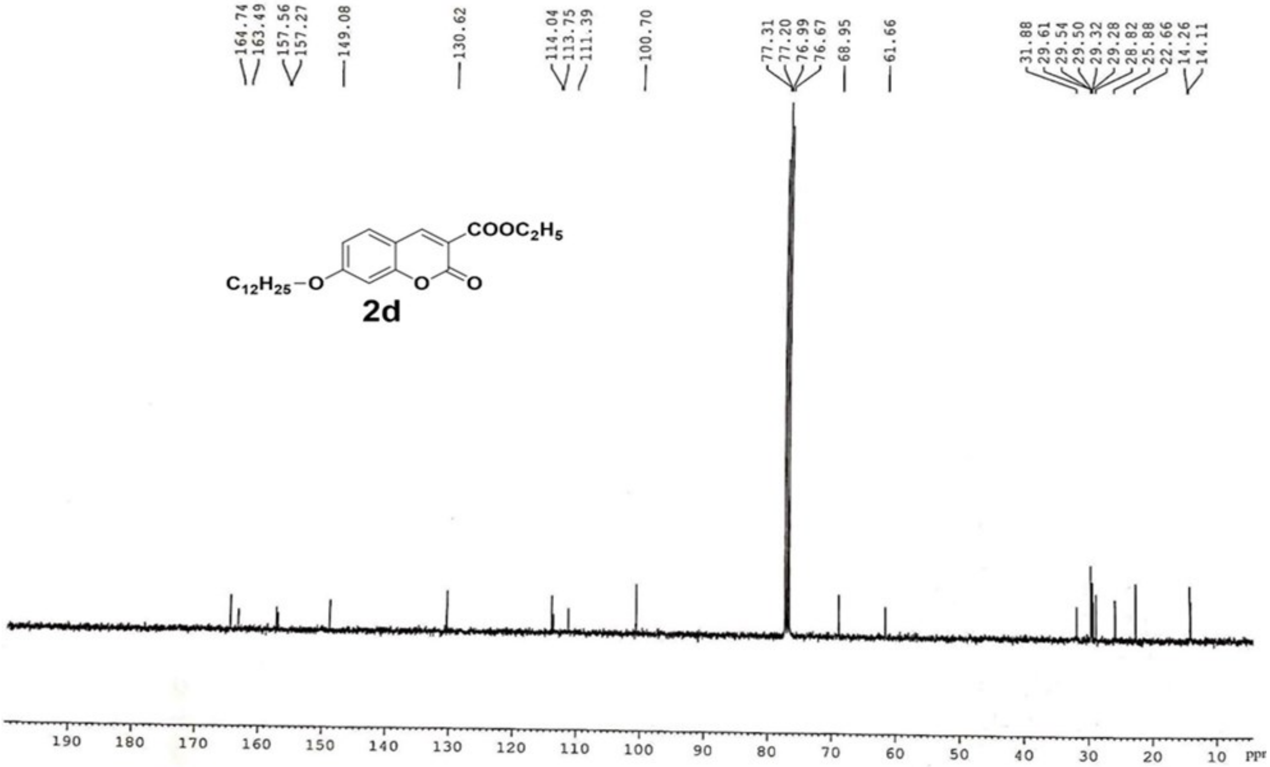

Figure 10: **^{13}C NMR spectrum of 2d (100 MHz, $CDCl_3$)**

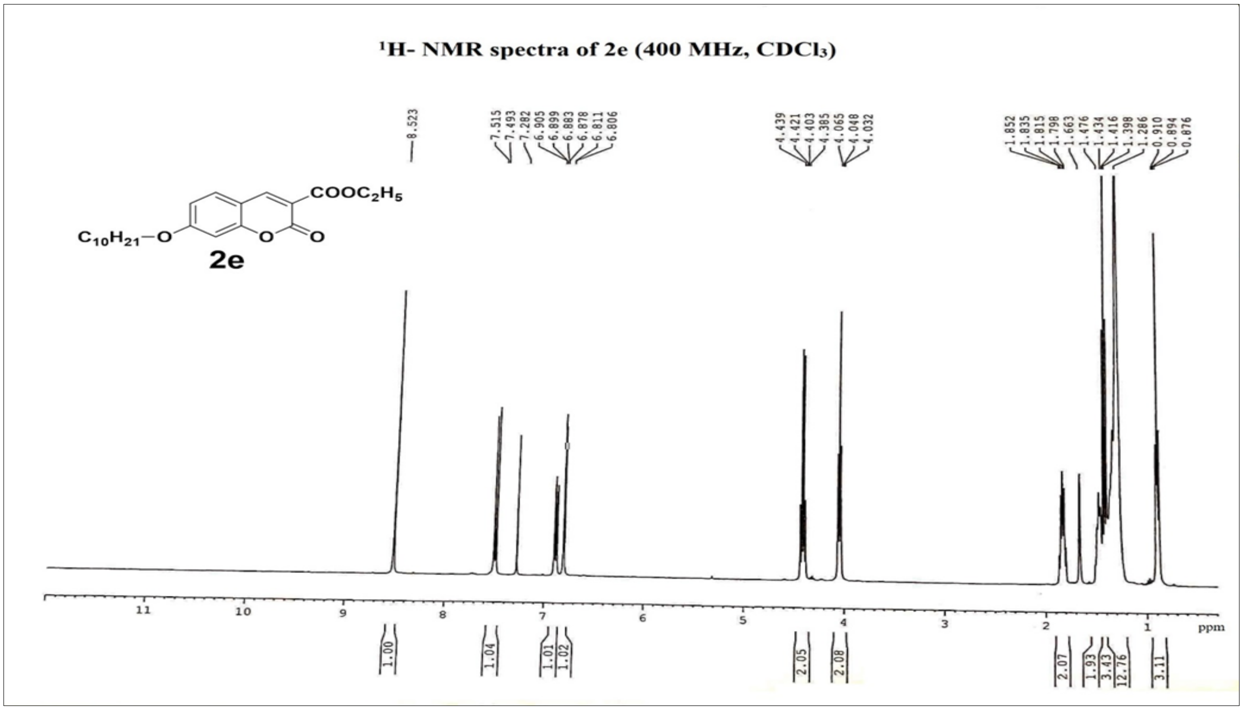

Figure 11: **^{1}H NMR spectrum of 2e (400 MHz, $CDCl_3$)**

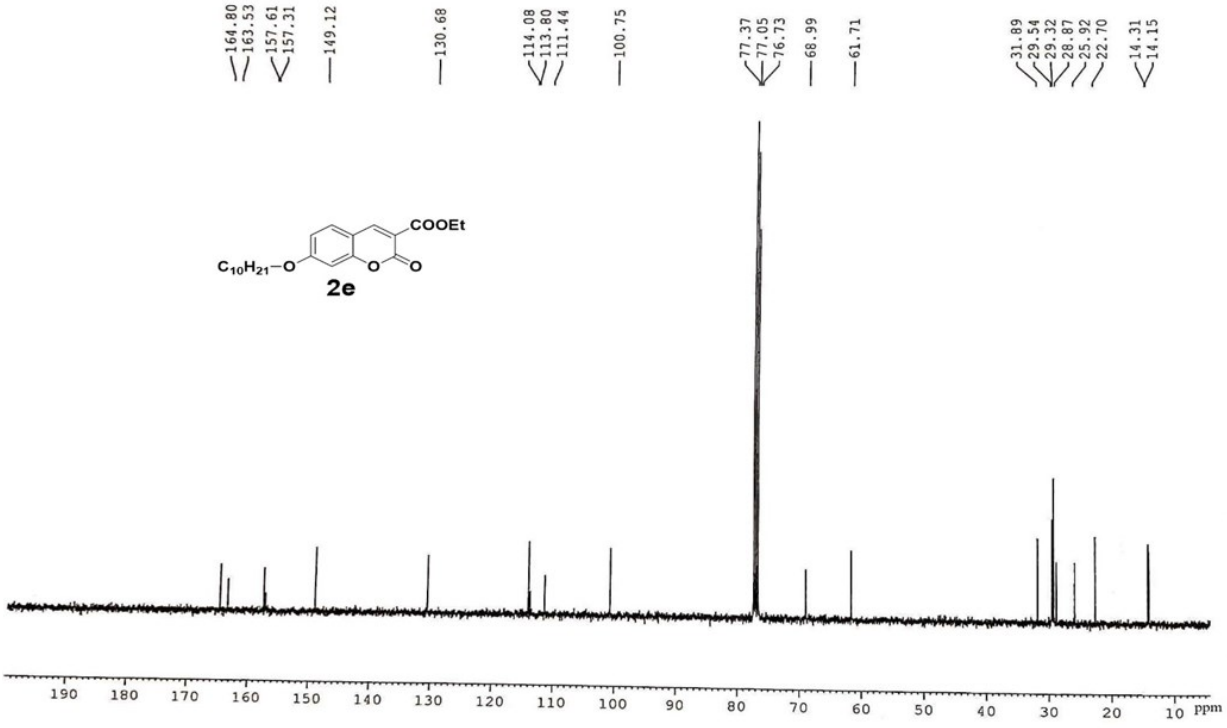

Figure 12: **^{13}C NMR spectrum of 2e (100 MHz, $CDCl_3$)**

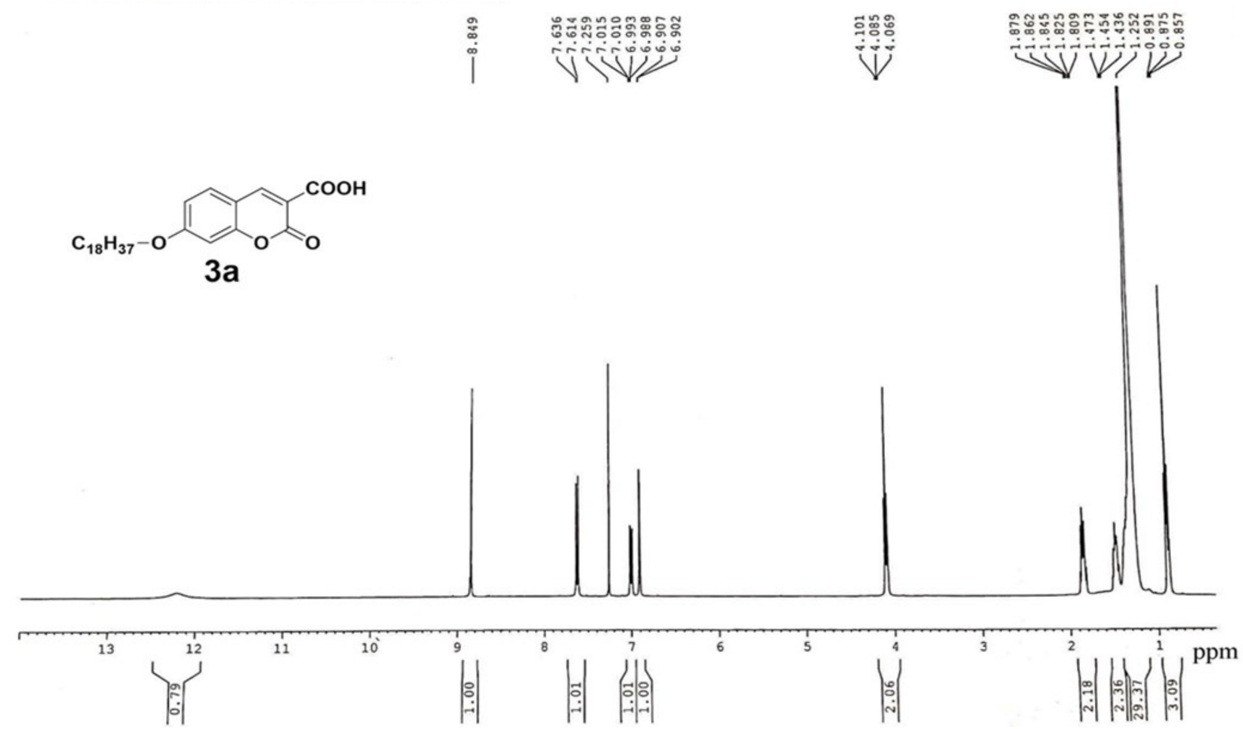

Figure 13: **^{1}H NMR spectrum of 3a (400 MHz, $CDCl_3$)**

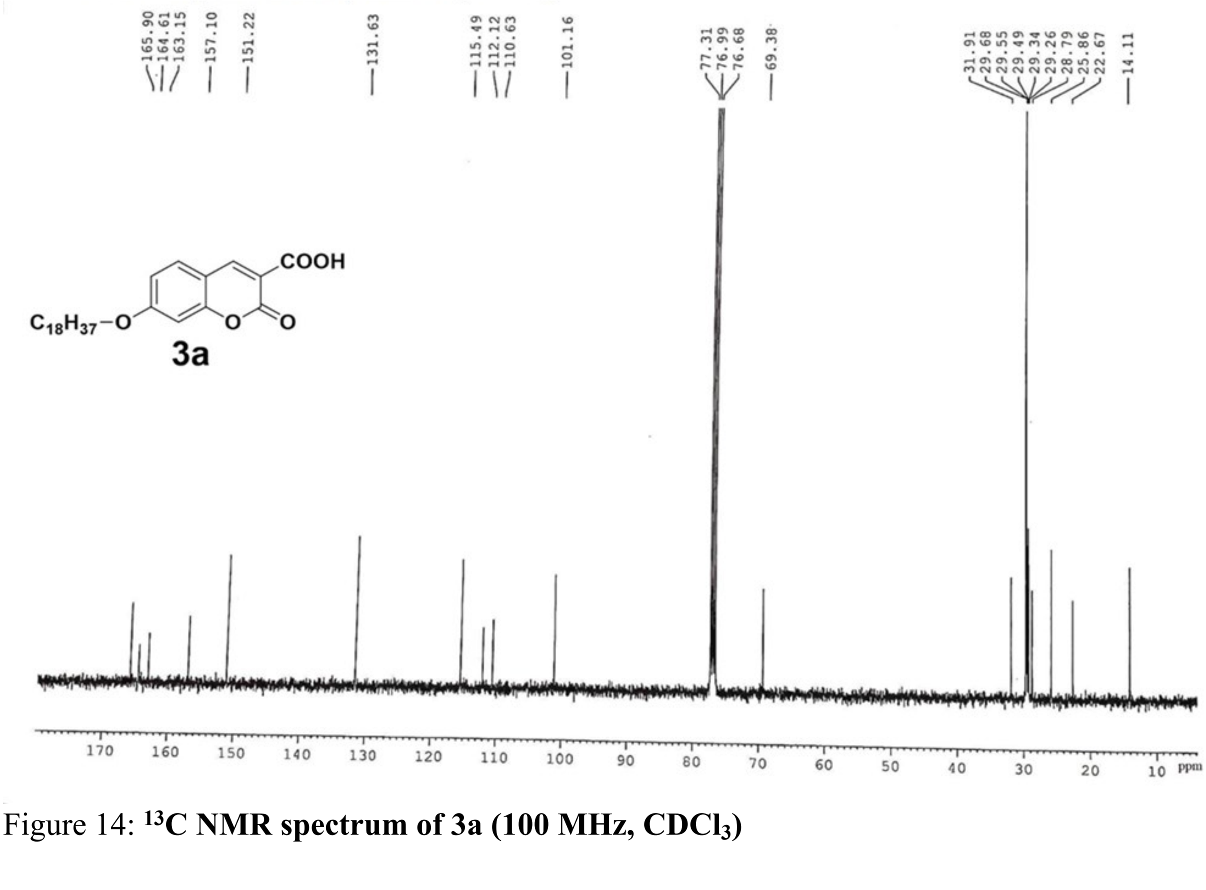

Figure 14: **^{13}C NMR spectrum of 3a (100 MHz, $CDCl_3$)**

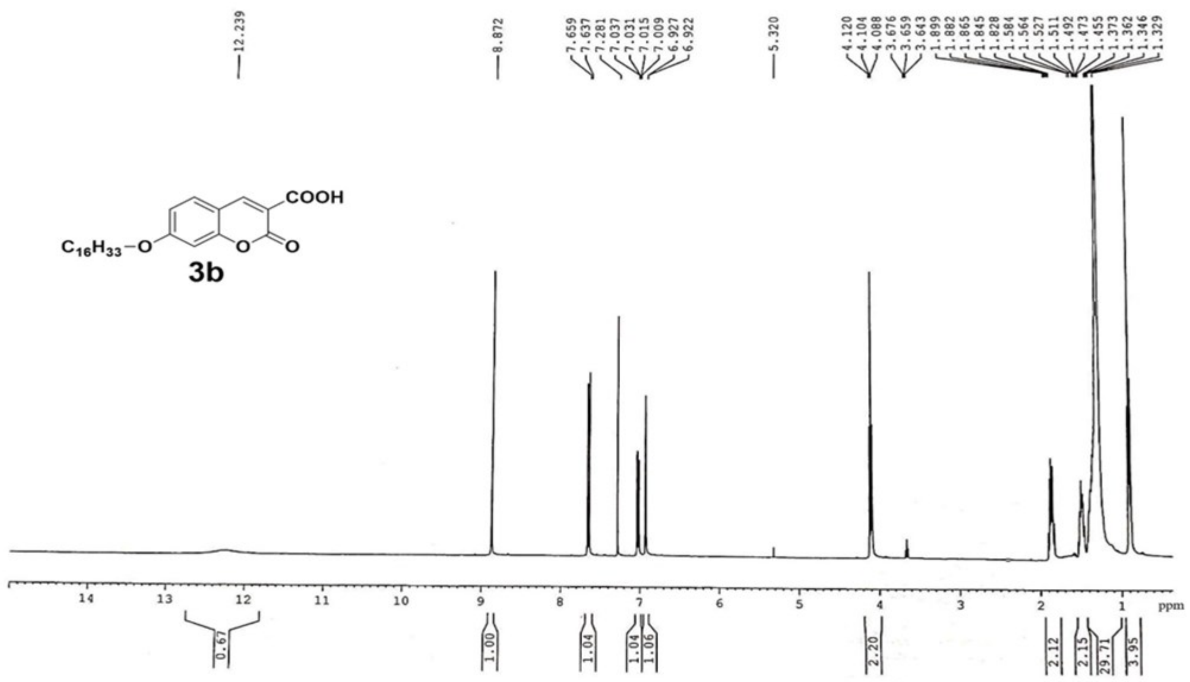

Figure 15: **^{1}H NMR spectrum of 3b (400 MHz, $CDCl_3$)**

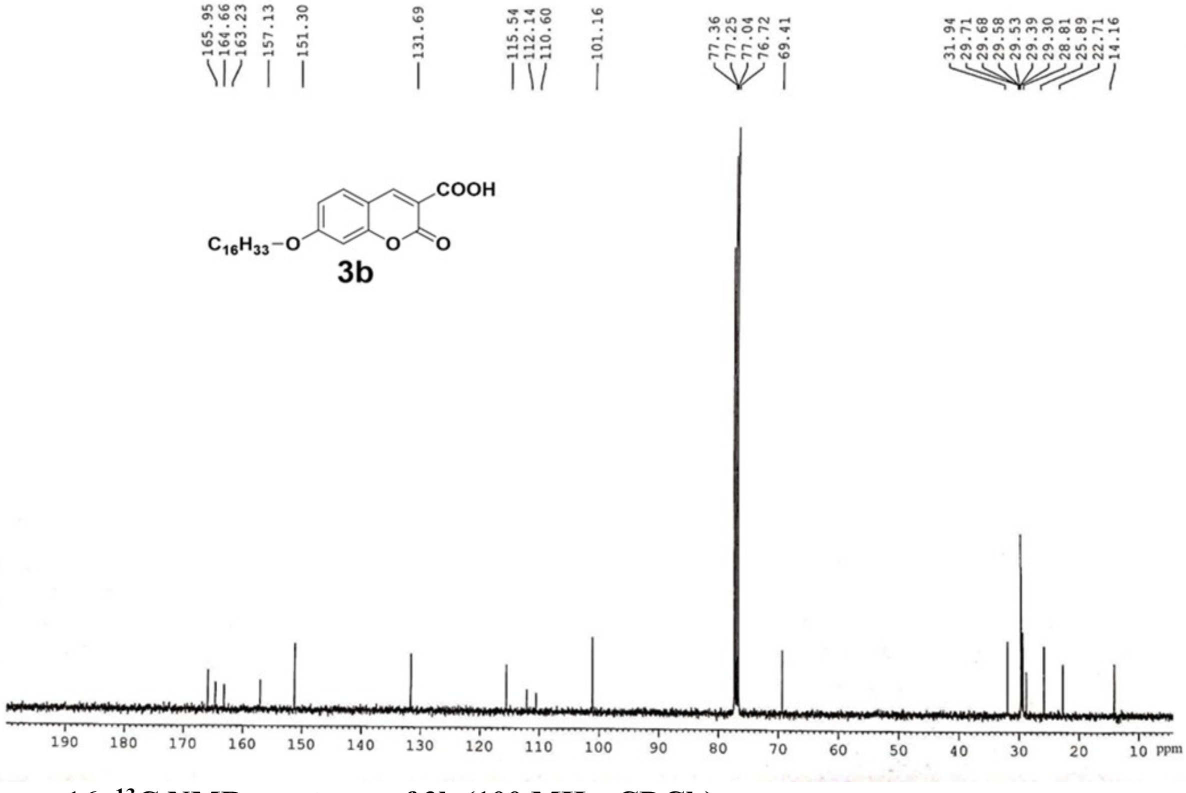

Figure 16: **^{13}C NMR spectrum of 3b (100 MHz, $CDCl_3$)**

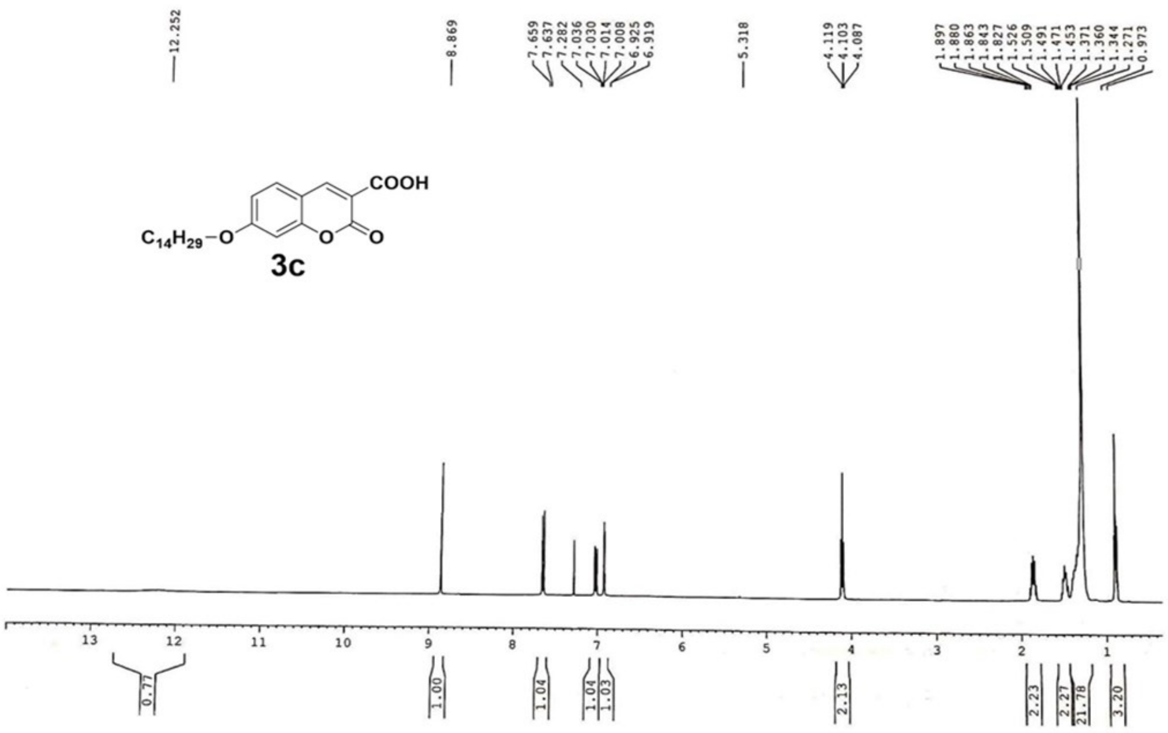

Figure 17: **^{1}H NMR spectrum of 3c (400 MHz, $CDCl_3$)**

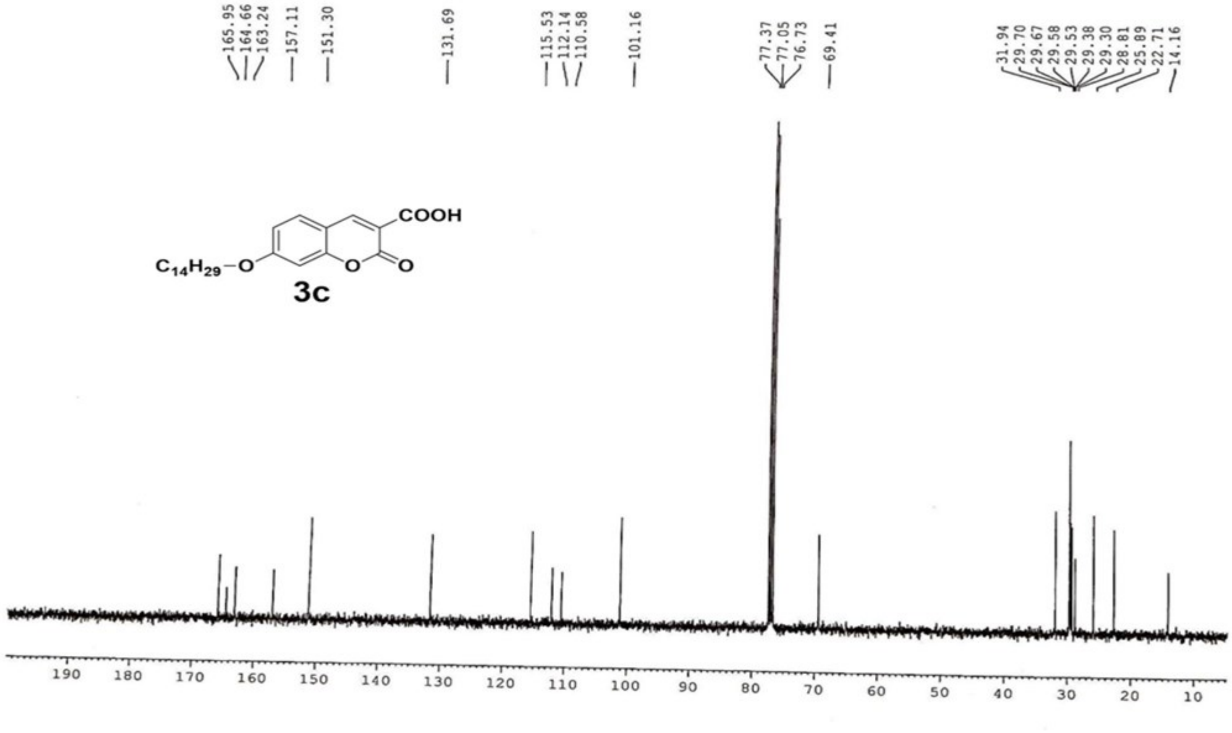

Figure 18: **^{13}C NMR spectrum of 3c (100 MHz, $CDCl_3$)**

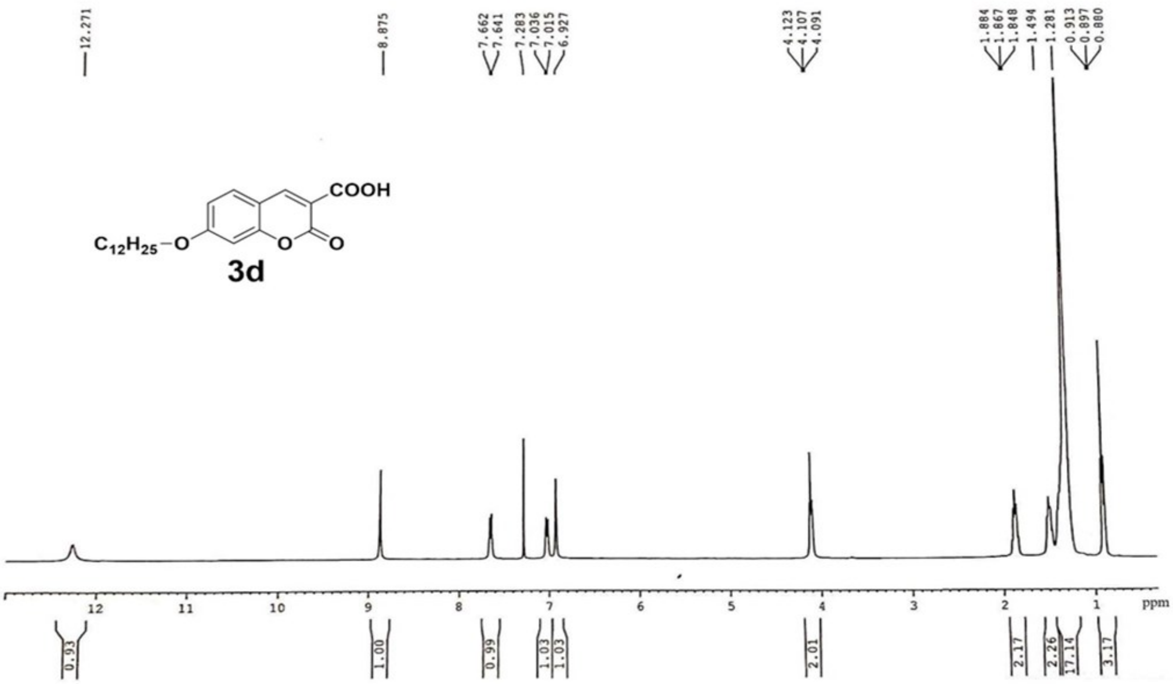

Figure 19: **^{1}H NMR spectrum of 3d (400 MHz, $CDCl_3$)**

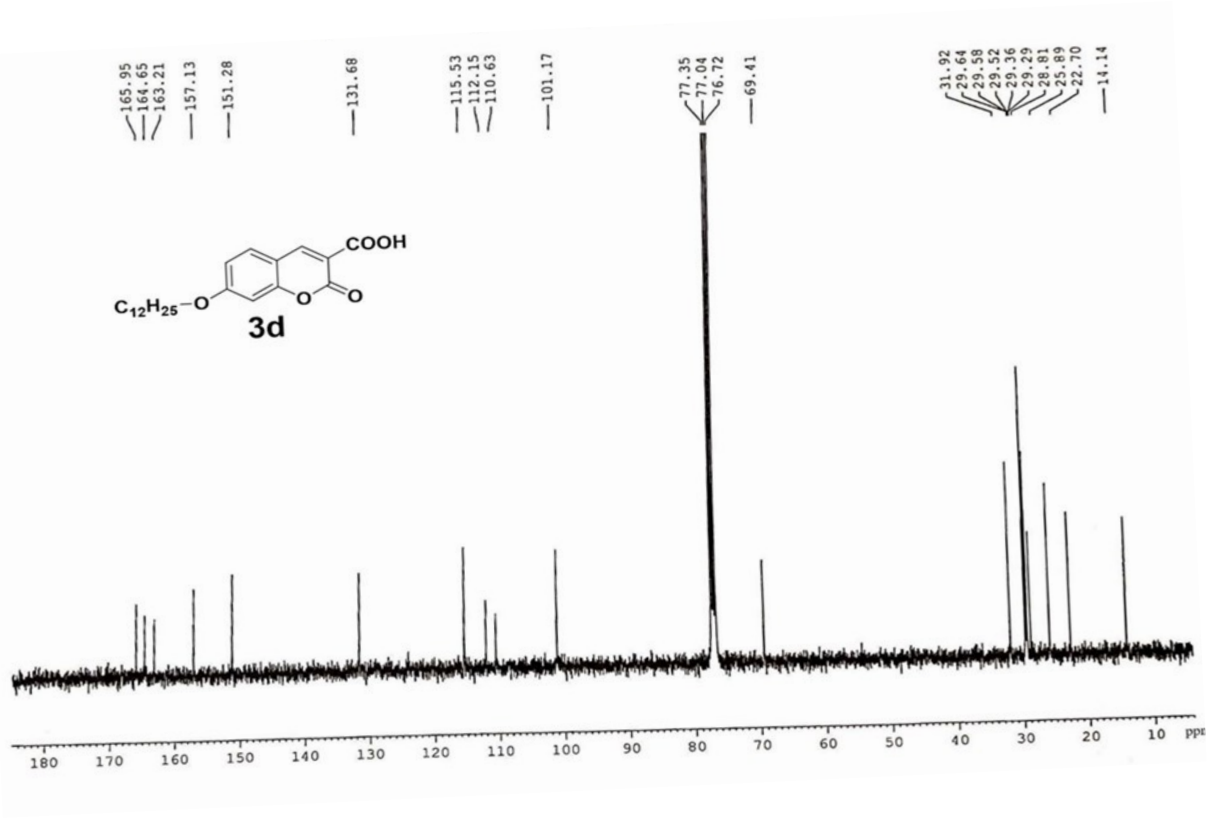

Figure 20: **^{13}C NMR spectrum of 3d (100 MHz, $CDCl_3$)**

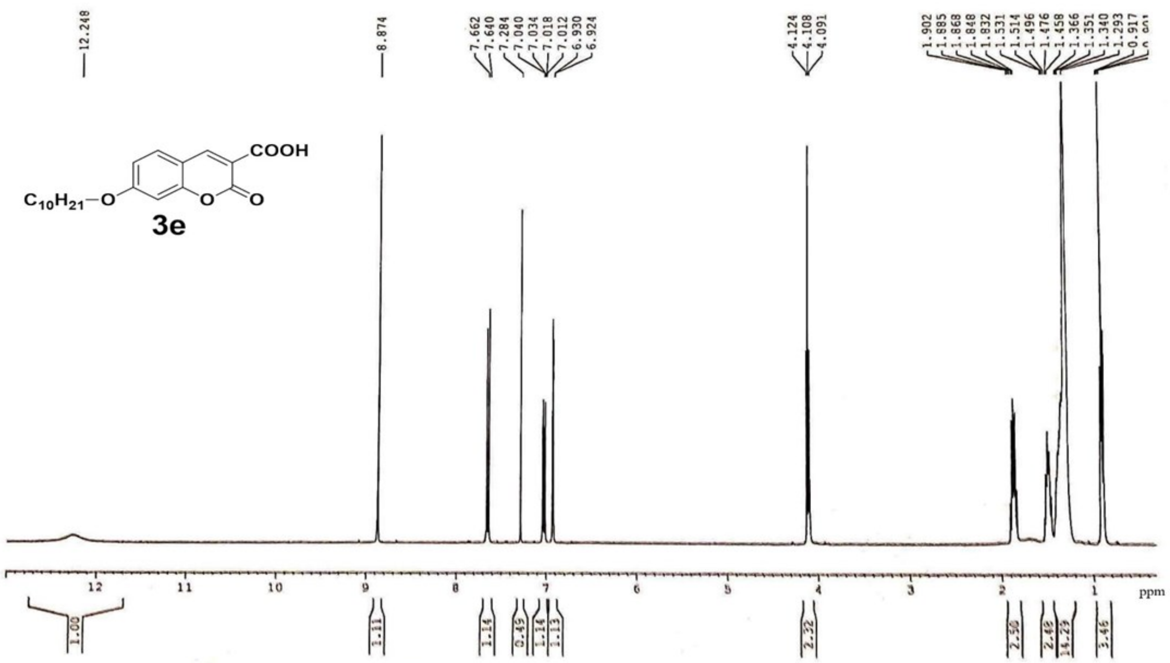

Figure 21: **^{1}H NMR spectrum of 3e (400 MHz, $CDCl_3$)**

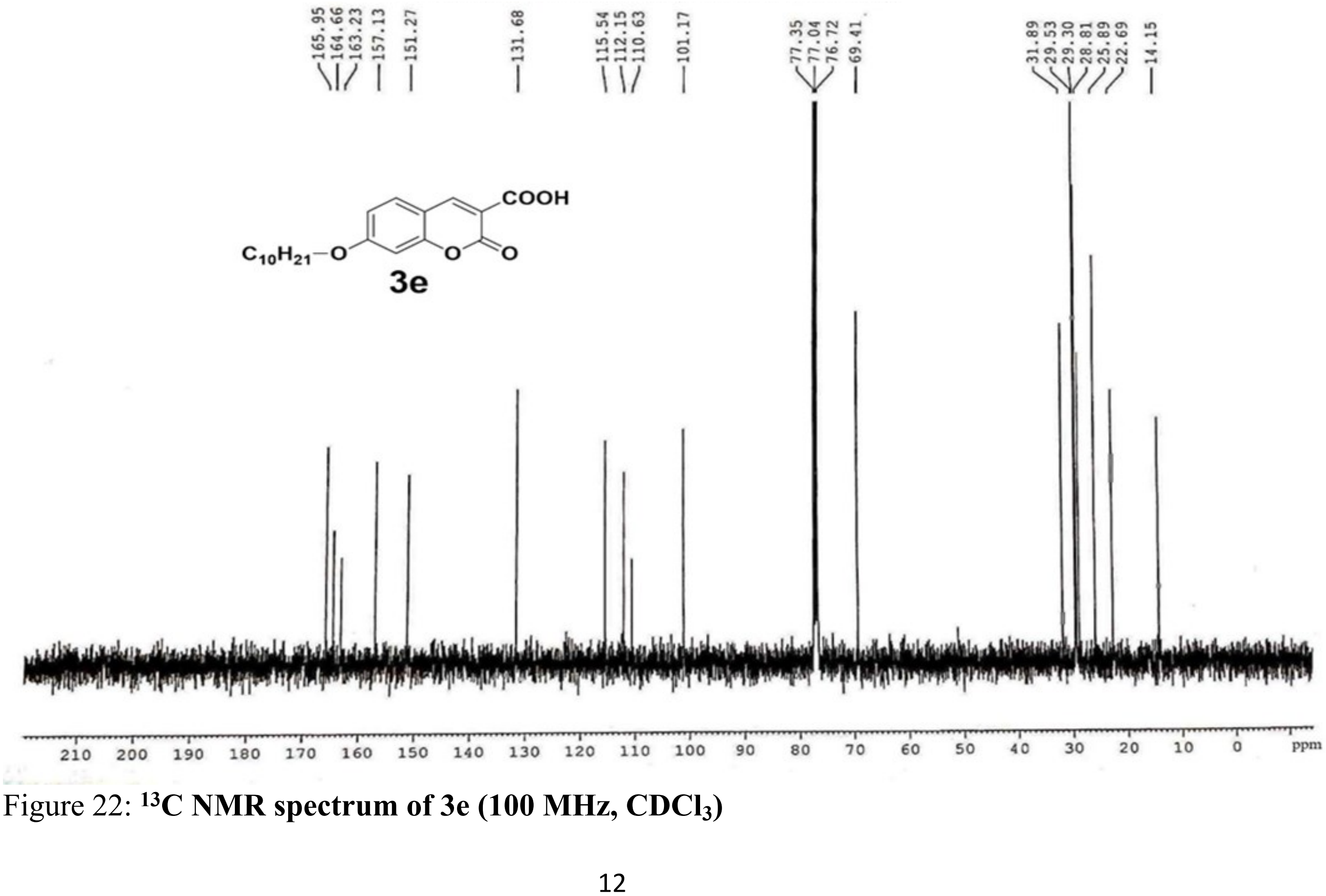

Figure 22: **^{13}C NMR spectrum of 3e (100 MHz, $CDCl_3$)**

Fluorescence spectra

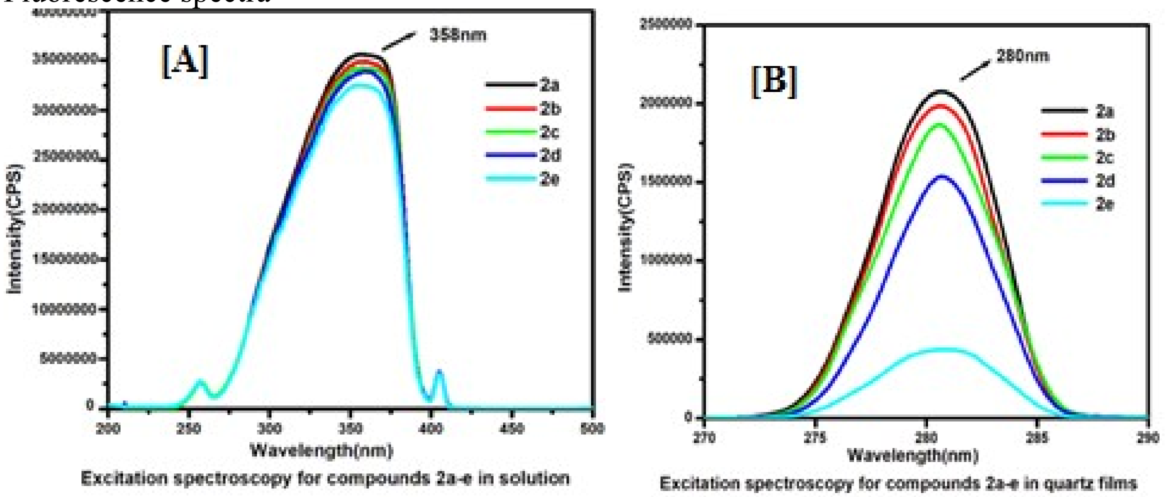

Figure 23a: Excitation spectra for compounds **2a-e** in solution [A] and films [B]

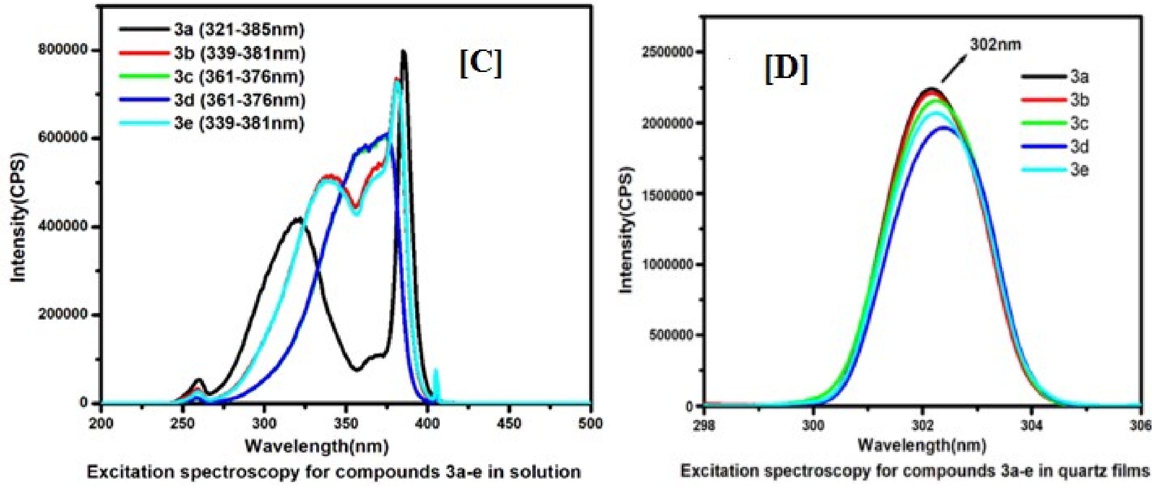

Figure 23b: Excitation spectra for compounds **3a-e** in solution [C] and films [D]

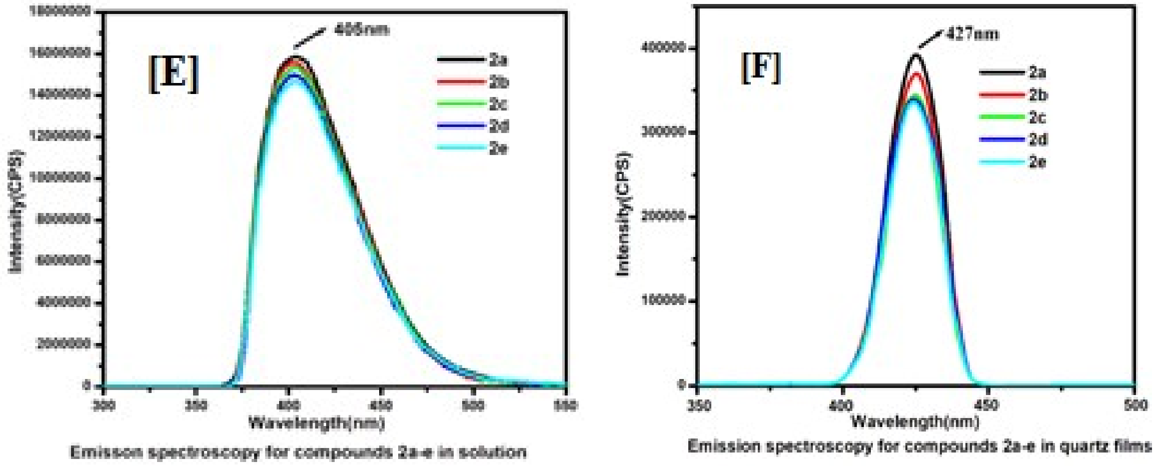

Figure 23c: Emission spectra for compounds **2a-e** in solution [E] and films [F]

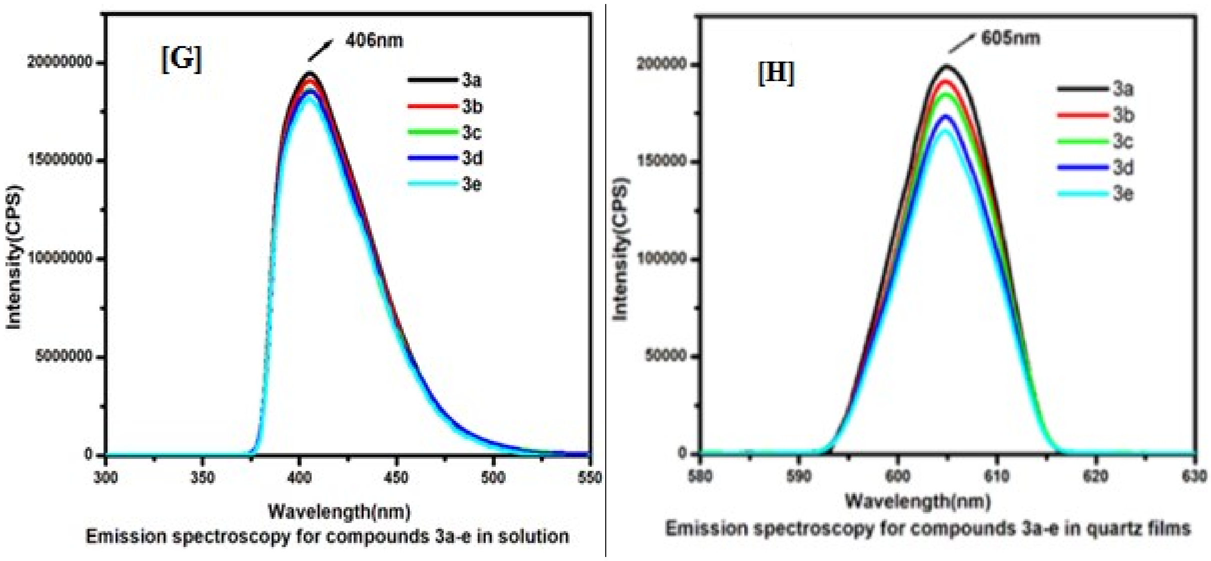

Figure 23d: Emission spectra for compounds **3a-e** in solution [G] and films [H]

Figure 23: Fluorescence spectra for compounds (**2a-e**) and (**3a-e**) in solution ($CHCl_3$) and spin coated films.

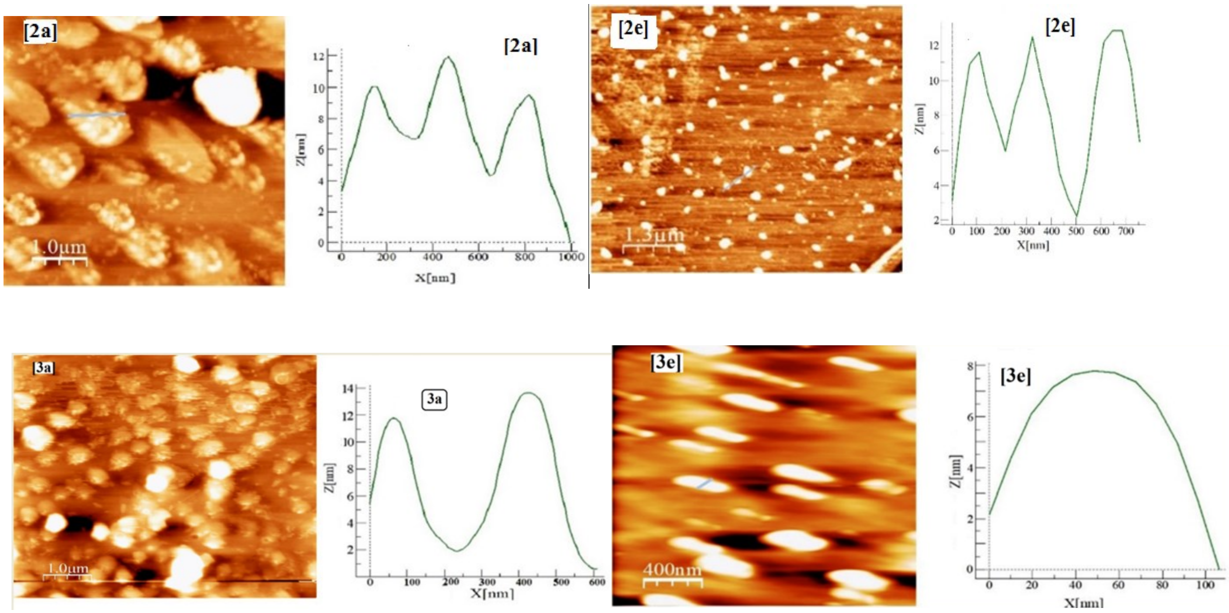

Figure 24: AFM images of thin films for compounds **2a, 2e, 3a** and **3e** onto the silicon substrate
